# Structure-guided development of a high-affinity human Programmed Cell Death-1: Implications for tumor immunotherapy

**DOI:** 10.1016/j.ebiom.2017.02.004

**Published:** 2017-02-06

**Authors:** Eszter Lázár-Molnár, Lisa Scandiuzzi, Indranil Basu, Thomas Quinn, Eliezer Sylvestre, Edith Palmieri, Udupi A. Ramagopal, Stanley G. Nathenson, Chandan Guha, Steven C. Almo

**Affiliations:** a Department of Microbiology & Immunology, Albert Einstein College of Medicine, 1300 Morris Park Ave, Bronx, NY 10461, USA; b Department of Radiation Oncology, Albert Einstein College of Medicine, 1300 Morris Park Ave, Bronx, NY 10461, USA; c Department of Biochemistry, Albert Einstein College of Medicine, 1300 Morris Park Ave, Bronx, NY 10461, USA; d Department of Cell Biology, Albert Einstein College of Medicine, 1300 Morris Park Ave, Bronx, NY 10461, USA; e Department of Pathology, Albert Einstein College of Medicine, 1300 Morris Park Ave, Bronx, NY 10461, USA; f Department of Physiology & Biophysics, Albert Einstein College of Medicine, 1300 Morris Park Ave, Bronx, NY 10461, USA

**Keywords:** PD-1, Immunotherapy, High-affinity mutant, Radio-immunotherapy, PD-1 Ig fusion protein

## Abstract

Programmed Cell Death-1 (PD-1) is an inhibitory immune receptor, which plays critical roles in T cell co-inhibition and exhaustion upon binding to its ligands PD-L1 and PD-L2. We report the crystal structure of the human PD-1 ectodomain and the mapping of the PD-1 binding interface. Mutagenesis studies confirmed the crystallographic interface, and resulted in mutant PD-1 receptors with altered affinity and ligand-specificity. In particular, a high-affinity mutant PD-1 (HA PD-1) exhibited 45 and 30-fold increase in binding to PD-L1 and PD-L2, respectively, due to slower dissociation rates. This mutant (A132L) was used to engineer a soluble chimeric Ig fusion protein for cell-based and *in vivo* studies. HA PD-1 Ig showed enhanced binding to human dendritic cells, and increased T cell proliferation and cytokine production in a mixed lymphocyte reaction (MLR) assay. Moreover, in an experimental model of murine Lewis lung carcinoma, HA PD-1 Ig treatment synergized with radiation therapy to decrease local and metastatic tumor burden, as well as in the establishment of immunological memory responses. Our studies highlight the value of structural considerations in guiding the design of a high-affinity chimeric PD-1 Ig fusion protein with robust immune modulatory properties, and underscore the power of combination therapies to selectively manipulate the PD-1 pathway for tumor immunotherapy.

## Highlights

• We report the crystal structure of human PD-1 and the mapping of the PD-1 ligand binding site.
• A high-affinity variant was identified and used to engineer a soluble PD-1 Ig fusion protein (HA PD-1 Ig) for immunotherapy.
• HA PD-1 Ig enhanced T cell activation and strongly synergized with RT to control tumor growth in a lung carcinoma model.

PD-1 is an inhibitory immune receptor that dampens T cell responses. PD-1 blockade is a successful strategy for cancer immunotherapy.

We report the crystal structure of human PD-1 and mapping of its ligand binding site. A high-affinity mutant (HA PD-1) was identified and used to engineer a blocking Ig fusion protein, expected to disrupt the endogenous PD-L/PD-1 pathway.

HA PD-1 Ig increased lymphocyte proliferation and cytokine production. Moreover, in combination with radiation therapy it enhanced control of tumor growth in a Lewis lung carcinoma model. This study offers an alternative strategy to manipulate the PD-1 pathway for tumor immunotherapy.

## 1 Introduction

Programmed Cell Death-1 (PD-1) is an inhibitory immune receptor, which plays important roles in T cell co-inhibition and exhaustion, and is a prominent target for cancer immunotherapy. PD-1 was discovered in lymphoid murine cell lines induced to undergo apoptosis by PMA stimulation (Ishida et al., 1992, Okazaki and Honjo, 2007). Subsequent studies demonstrated that PD-1 expression is not sufficient to induce apoptosis; but it rather correlates with lymphocyte activation. The immune inhibitory role of PD-1 is consistent with the autoimmune phenotype observed in PD-1 deficient mice, with the type and severity of disease depending on the genetic background. C57BL/6 mice develop lupus-like arthritis and glomerulonephritis at over 6 months of age (50% penetrance), while Balb/C mice develop fatal dilated cardiomyopathy and die at ~ 4 weeks of age (Nishimura et al., 1999, Nishimura et al., 2001). Thus, the PD-1/PD-Ligand pathways have central roles in maintaining peripheral tolerance and protecting against autoimmunity. In humans, PD-1 polymorphisms are associated with susceptibility to autoimmune diseases, including systemic lupus erythematosus, diabetes, multiple sclerosis, rheumatoid arthritis and Grave's disease (Gianchecchi et al., 2013).

PD-1 is expressed on activated CD4^+^ and CD8^+^ T cells, as well as on B cells, myeloid cells (monocytes and some dendritic cells) and NK T cells, suggesting multiple roles in immune regulation (Keir et al., 2008). Persistent expression of PD-1 on CD8^+^ T cells leads to exhaustion, associated with decreased effector functions, including diminished secretion of IL-2, IFN-γ and TNF-α, and the inability to produce cytolytic molecules such as perforin (Hofmeyer et al., 2011). In addition, PD-1 is constitutively expressed on CD4^+^ Foxp3^+^ regulatory T cells (Tregs), regulating the development, maintenance and functional response of induced Treg cells, and facilitating *de novo* conversion of naïve CD4^+^ T cells to Foxp3^+^ Tregs (Francisco et al., 2010, Francisco et al., 2009). Thus, PD-1 makes broad contributions to T cell-mediated immunity by reducing effector T cell signaling and by enhancing immunosuppressive Treg function, which impacts the establishment and maintenance of immunological tolerance. Currently three monoclonal antibodies targeting PD-1 are under Phase I/III clinical trials for the treatment of various solid tumors, and two of them, pembrolizumab and nivolumab were granted FDA approval in 2014 for the treatment of metastatic melanoma (Sharma and Allison, 2015a), and subsequently for the treatment of advanced non-small cell lung cancer (NSCLC).

PD-1 recognizes two ligands, PD-L1 (B7-H1 or CD274) and PD-L2 (B7-DC, CD273), which belong to the B7 family and share 34% identity. PD-L1 mRNA is ubiquitously expressed by immune cells, as well as non-hematopoietic cells, and cell surface PD-L1 is upregulated upon activation. Cytokines such as IFN-γ and TNF-α induce PD-L1 expression on T and B cells, endothelial and epithelial cells (Freeman et al., 2000, Ishida et al., 2002). PD-L1 overexpression on tumor cells, and PD-1 on infiltrating lymphocytes have been recognized as important immune evasion mechanisms. Currently four different monoclonal PD-L1 blocking antibodies are in Phase I/II clinical trials (Ohaegbulam et al., 2015). PD-L2 exhibits ~ 3-fold higher affinity to PD-1 than PD-L1, but its expression is primarily restricted to antigen presenting cells such as dendritic cells, macrophages, B1 B cells and mast cells (Keir et al., 2008). PD-L2 expression is upregulated by cytokines such as IL-4 and IFN-γ (Latchman et al., 2001, Tseng et al., 2001, Zhong and Rothstein, 2011). Due to the broader expression of PD-1 and its ligands, compared to other costimulatory receptor-ligand pairs (such as CTLA4/B7, which are restricted to T cells/APCs), PD-1 signaling regulates immune responses at multiple levels, including, but not limited to, effector responses at the level of peripheral cells and tissues.

PD-1 and its ligands are single-pass type I transmembrane proteins, similar to other members of the CD28/B7 family (Chattopadhyay et al., 2009). PD-1 consists of an extracellular immunoglobulin variable (IgV) domain, a transmembrane segment and a cytoplasmic tail harboring two tyrosine-based signaling motifs. The ectodomains of the PD-Ligands are composed of membrane-distal IgV and membrane-proximal immunoglobulin constant (IgC) domains, followed by transmembrane and cytoplasmic segments. The human and mouse PD-1 genes share 60% and 70% identity at the amino acid and nucleotide levels, respectively (Finger et al., 1997). Binding of the PD-Ligands to PD-1 in the context of antigen receptor signaling induces phosphorylation of the two signaling tyrosines within the cytoplasmic tail of PD-1, one of which is part of an Immunoreceptor Tyrosine-based Inhibitory Motif (ITIM), and the other an Immunoreceptor Tyrosin-based Switch Motif (ITSM). Src homology 2-containing tyrosine phosphatase (SHP-2) is recruited to the phosphorylated ITSM motif, which dephosphorylates signaling molecules such as TCR-associated CD3ζ and ZAP70, resulting in inhibition of the downstream PI3K/Akt signaling pathway, and disruption of glucose metabolism and IL-2 production in T cells (Keir et al., 2005, Okazaki et al., 2001, Sharpe et al., 2007).

Recent studies demonstrate that PD-1 is a crucial regulator of immune responses against microbial pathogens (Lazar-Molnar et al., 2010, Lazar-Molnar et al., 2008a). Persistent expression of PD-1 on CD8^+^ T cells in chronic viral infections, such as LCMV (Barber et al., 2006), HIV (Day et al., 2006) and hepatitis (Urbani et al., 2006), has been linked to T cell exhaustion and unfavorable disease progression. Blockade of the PD-1 pathway can increase the effectiveness of anti-pathogen immune responses (Sharpe et al., 2007), making PD-1 an attractive candidate for immunotherapy of chronic infections.

We previously determined the crystal structure of the murine PD-1/PD-L2 complex (Lazar-Molnar et al., 2008b), and Garboczi's group reported the structure of the complex between mouse PD-1 and human PD-L1 (Lin et al., 2008). Structures of the unliganded murine and human PD-1 extracellular domains determined by crystallography (Zhang et al., 2004) and NMR (Cheng et al., 2013), respectively, have also been reported. Furthermore, structures of complexes between murine PD-1 mutants with human PD-L1 (pdb ID 3SBW), as well as with murine PD-L2 (pdb ID-s 3RNQ, 3RNK) have been determined, and most recently the crystal structure of human PD-1 in complex with human PD-L1 was reported (Zak et al., 2015).

Here we report the identification and biochemical, structural and functional characterization of a high-affinity mutant of human PD-1 variant (A132L) exhibiting approximately 45- and 30-fold higher affinities to PD-L1 and PD-L2, respectively. A soluble chimeric Ig fusion construct of this high-affinity PD-1 variant (HA PD-1 Ig) significantly increased proliferation and cytokine production of human T cells in an *in vitro* allogeneic mixed lymphocyte reaction (MLR). Notably, in an *in vivo* model of murine Lewis lung carcinoma, HA PD-1 Ig treatment in combination with radiation therapy (RT), synergistically decreased local and metastatic tumor burden, increased survival and induced immunological memory responses towards tumor re-challenge. These studies demonstrate that our structure-guided HA PD-1 Ig variant, in combination with stereotactic radiotherapy, represents a promising strategy for tumor immunotherapy.

## 2 Materials & Methods

### 2.1 Cloning, Expression, and Purification of Human PD-1 Extracellular Domain

The extracellular IgV domains of wild type and mutant human PD-1 (from W32 through P160; numbering relative to the initiator methionine) were cloned into the pET3a vector between the NdeI and BamH1 restriction sites. Expression plasmids were transformed into *E. coli* Rosetta 2 (DE3) pLysS strain (Novagen). Wild type and mutant human PD-1 inclusion bodies were purified and refolded as described for mouse PD-1 (Zhang et al., 2004). The refolding buffer was composed of 0.4 M Arginine-HCl, 100 mM Tris (pH 8.5), 2 mM EDTA, 5 mM cysteamine, and 0.5 mM cystamine; the refolded protein was purified by size exclusion chromatography (SEC) using a Superdex 75 column (GE Healthcare).

### 2.2 Crystallization, Data Collection, and Structure Determination

Crystals of human PD-1 A132L mutant were obtained by sitting drop vapor diffusion at 293 K, using 3.5 M sodium formate, 0.1 M Bis-Tris (pH 7.5) as the precipitant. Data were collected at the National Synchrotron Light Source (NSLS) beamline X-29A, and were consistent with the hexagonal space group *P* 6_5_22 (a = b = 46.02 Å, c = 187.40 Å, with a single molecule in the asymmetric unit). Data were integrated and scaled with HKL2000 (Otwinowski and Minor, 1997). The human A132L mutant PD-1 structure was determined by molecular replacement using the mouse PD-1 structure (1NPU) as the search model, followed by iterative cycles of manual rebuilding with COOT (Emsley and Cowtan, 2004) and crystallographic refinement with program REFMAC5 (Murshudov et al., 2011). The final model contains human A132L PD-1 residues 32–160, and 35 water molecules, resulting in R_work_/R_free_ of 21.4%/24.9%. Data collection and refinement statistics are summarized in Supplementary Table 1. Stereochemical parameters of the structure were evaluated with PROCHECK (Laskowski et al., 1993). Superpositions were calculated using COOT. Pymol was used for the preparation of structure figures (DeLano Scientific LLC). The atomic coordinates and reflections have been deposited in the Protein Data Bank under accession code 3RRQ.

### 2.3 Mutagenesis of Human PD-1

Mutants of full-length human PD-1 for mammalian expression were generated by PCR-based mutagenesis, using the Gateway pEF5/FRT/V5-DEST destination vector (Life Technologies). PD-1 constructs were transiently transfected into HEK293 cells using either Lipofectamine2000 (Life Technologies), or calcium phosphate transfection. Residues were selected for mutagenesis based on similarity with mouse PD-1, and analysis of the mouse PD-1/PD-L2 interface reported earlier (Lazar-Molnar et al., 2008b). Full-length wild type human and mouse PD-L1 and PD-L2 were cloned into the EYFP-N1 expression vector (Clontech), and expressed as C-terminal YFP fusion proteins in HEK293 cells.

### 2.4 Ig Fusion Proteins and Antibodies

Human and mouse PD-L1 and PD-L2 Ig fusion proteins were purchased from R&D Systems, and characterized by SEC prior to use. Functional grade PD-L1 blocking antibodies was purchased from Affymetrix eBioscience (clone MIH1) and from BioXCell (clone 10F.9G2). Human CD11c-FITC and MHCII-APC antibodies were purchased from Life Technologies; CD14-FITC, PD-1-FITC (MIH4 and J116), PD-L1-PE-Cy7, PD-L2-APC, CD83-APC and B7-1-FITC antibodies from Affymetrix eBioscience.

### 2.5 Surface Plasmon Resonance Binding Assays

Surface Plasmon Resonance (SPR) binding experiments were performed with a BIAcore 3000 optical biosensor at 25 °C. Murine or human PD-L1-Ig or PD-L2-Ig fusion proteins were immobilized on the surface of a CM5 sensor chip by free amine coupling, followed by deactivation with ethanolamine (following the manufacturer's protocol). For determination of equilibrium dissociation constants (K_d_), a range of concentrations (0.01–100 μM) of PD-1 or its mutants were injected sequentially over the flow cell at a rate of 20 μl/min. K_d_s were obtained by fitting the data to the non-linear 1:1 Langmuir binding model, using the relationship: R = R_max_C / (C + K_d_), where R is the bound analyte in response units (RU), R_max_ is the maximum response level, C is the concentration of the free analyte in μM and K_d_ is in μM.

### 2.6 Expression and Purification of Wild Type and Mutant PD-1 Ig

The ectodomain of human PD-1 was fused to the Fc domain of human IgG1 followed by C-terminal 6xHis tag, and the endogenous PD-1 signal peptide was replaced by β2-microglobulin signal peptide. Mutant PD-1 Ig fusion proteins (A132L, L128R and K78A) were generated by PCR-based mutagenesis. The fusion constructs were cloned into the pIRES2-EGFP expression vector (Clontech) and transfected into HEK293 cells using calcium phosphate. Stable cell lines were generated by antibiotic selection (G418, 1 mg/ml) and FACS sorting. Ig fusion proteins were purified from cell culture supernatants by affinity chromatography using Ni^2 +^-NTA Agarose (Qiagen). The proteins were eluted with 250 mM imidazole (pH = 8) and exchanged into 1xPBS. The purity and quality of the eluted protein was assessed by SDS-PAGE and SEC.

### 2.7 Flow Cytometric Binding Assays

HEK293 cells transiently transfected with wild type or mutant PD-1 constructs were incubated with PD-L1 or PD-L2 Ig fusion proteins (0.01–50 μg/ml) for one hour on ice. After washing, the cells were further incubated for 30 min in the presence of APC-conjugated goat anti-human IgG F(ab′)_2_, Fcγ fragment specific (Jackson Immunoresearch). After washing, the cells were fixed in 2% paraformaldehyde. Samples were processed using LSRII or FACS Canto II flow cytometers (BD Biosciences), and analyzed using Flow Jo software (Tree Star, Inc.). Mean Fluorescence Intensities were normalized to wild type PD-1 binding. Cell surface expression of PD-1 constructs was quantified by monoclonal antibody staining.

For binding experiments using human PD-1 Ig proteins, differentiated human dendritic cells, or PD-L1 or PD-L2 transfected HEK293 cells were incubated with the Ig fusion proteins, followed by incubation with Cy5-conjugated goat anti-human IgG F(ab′)_2_ (Jackson Immunoresearch). The expression of the PD-L1 and PD-L2 ligands on the cell surface was verified by monoclonal antibody staining.

### 2.8 Dendritic Cell Differentiation and MLR Assay

PBMCs from the peripheral blood of healthy donors were isolated by Ficoll gradient centrifugation. Monocytes were enriched by plastic adherence for 2 h. After removing non-adherent cells, adherent cells were cultured for 5 days in the presence of GM-CSF 100 ng/ml and IL-4 50 ng/ml (R&D Systems) to induce formation of immature dendritic cells. Fresh cytokines were added every 3 days. On day 6 of culture, 100 ng/ml TNF-α was also added to promote the formation of “mature” dendritic cells. These cells were used for subsequent binding and functional assays. Human CD4^+^ T cells from PBMCs of healthy donors were purified by negative selection, using EasySep kit (Stemcell Technologies).

For allogeneic stimulation, freshly purified human T cells (2 × 10^5^/well) were co-cultured with the differentiated dendritic cells derived from an allogeneic donor, using 16:1 T cell: DC ratios. Prior to adding to the cultures, DCs were irradiated using 2000 rad, and incubated with various concentrations of wild type and mutant human PD-1 Ig fusion proteins for 30–60 min. After addition of T cells, allogeneic stimulation proceeded for 5 days before addition of 1 mCi/well of 3H-thymidine (Perkin Elmers) for the final 12 h of incubation. Culture supernatants collected at day 4 were assayed for multiple cytokines using FlowCytomix Multiplex kits Th1/Th2 11-plex, or Th1/Th2/Th17 13-plex (Affymetrix eBioscience).

### 2.9 Mice and Tumor Cell Line

6-week-old female C57BL/6 mice were purchased from Jackson Laboratory. All mice were maintained under specific pathogen-free conditions. The murine Lewis lung carcinoma (3LL) cell line was purchased from the American Type Culture Collection (ATCC) and cultured in DMEM (Gibco) supplemented with 10% FBS and 250 IU of penicillin/streptomycin.

### 2.10 Tumor Growth

Primary tumors were established by subcutaneous injection of 10^5^ 3LL cells (in 30 μl) subcutaneously into the dorsum of the right hind limb (Fig. 4A). Once primary tumor volumes reached 40–80 mm^3^, mice were randomized into treatment groups. Tumor growth in the foot and in draining lymph nodes was monitored by measuring tumor size with Vernier calipers three times per week. Tumor volume was calculated using an ellipsoid formula: V = (π/6 × length × width × height). Primary dorsal hind limb tumors exhibit Gompertzian growth, with a phase I volume of 40–80 mm^3^, phase II volume of 150–300 mm^3^, and phase III volume of > 300–500 mm^3^. Therefore, treatment efficacy was evaluated by determining the tumor growth delay (TGD) to quadruple the initial volume (*V*_e_) according to: *t*_e_ = *t*_1_ + (*t*_2_ − *t*_1_)log(*V*_e_/*V*_1_)/log(*V*_2_/*V*_1_), where *V*_1_ and *V*_2_ are measured volumes that flank *V*_e_ at times *t*_1_ and *t*_2_, respectively. Tumors ≥ 300–500 mm^3^ have reached phase III growth due to anatomical and vascular limitations, and below-the-knee amputations were performed on mice for the metastases studies, in accordance with our IACUC approved protocol.

### 2.11 Metastasis Evaluation

Isolated lungs from mice receiving below-the-knee amputation (as previously described) were injected intratracheally using 1 mL of Fekete's solution (ethanol, glacial acetic acid and formaldehyde based fixative) to insufflate the lobes. The trachea was clamped, and the entire lung and heart removed *en bloc* and washed with PBS. Lungs were submerged and fixed in Fekete's solution for 24 h, followed by transfer to 4% paraformaldehyde prior to enumeration of pulmonary metastases. The left lung and the 4 lobes of the right lung were isolated and nodules counted under a dissecting microscope.

### 2.12 Tumor Re-Challenge and Analysis of Memory Response

Fifty-seven days after tumor treatment, cured mice were reinjected on the opposite foot with the same number of cells used for the first tumor inoculation (1 × 10^5^ 3LL tumor). Naïve mice were also injected as controls. Mice were monitored until day 66 and then sacrificed to evaluate immune memory T cell populations (T_EM,_ CD44^hi^CD62L^lo^, T_CM_, CD44^hi^CD62L^hi^) in spleen and draining lymph nodes. The following antibodies were used: CD62L-PE clone MEL-14, CD44-FITC clone IM7 from eBioscience; CD45-Alexa 700 clone 30-F11, CD3-Pe-Cy5 clone 145-2C11, PD-L1-BV421 clone MIH-5 from BD Bioscience; CD4-APC-Cy7 clone RM4-5, CD8-PerCP clone 53–67 from Biolegend. Cells were acquired on a BD LSR-II Flow Cytometer and analyzed using the FlowJo software (Tree Star, Inc.).

### 2.13 Radiation Therapy and Treatments

All radiation was delivered using Xstrahl's Limited Small Animal Radiation Research Platform (SARRP). Briefly, a motorized stage and a constant voltage 225 kV X-ray tube, mounted on a motorized gantry, were controlled via a SARRP desktop application. Treatment plans were designed with 5 degrees of motion: translation (x, y, z) and rotation of animal stage, and by independent gantry rotation. Image-guided radiation therapy (IGRT) was performed using the SARRP's on-board cone beam CT (CBCT). CBCT imaging is achieved using a 1 mm aluminum filter with 50 kV, 0.7 mA, and a 20 × 20 cm flat panel detector for acquisition. Following CBCT acquisition, the treatment plan was constructed using 3D Slicer, an open-source software package (http://www.slicer.org). For radiation delivery, a 1 mm copper filter was fitted with a 10 × 10 mm collimator at a source-skin distance (SSD) of 35 cm, energized at 220 kV and 13.0 mA. Mice were anesthetized using a continuous flow of 1.5% isoflurane in pure oxygen at a rate of 1.5 L/min. Mice were placed in a left lateral decubitus position on the stage, attached to a motorized platform, and the tumor-bearing right hindlimb was extended, elevated, and secured to a 1.5 cm adhesive platform to minimize extraneous tissue exposure. 20 Gy was delivered over each of three successive days for a total hypofractionated dose of 60 Gy.

Mice were randomly distributed into the following treatment groups: 1) Control group receiving i.p. injection of 0.2 mg human IgG isotype control every 3 days for a total of 5 doses; 2) HA PD-1 Ig group, 3) mPD-L1 Ab group, injected i.p. with 0.2 mg HA PD-1 Ig, or mPD-L1 Ab, respectively, every 3 days for a total of 5 doses; 4) RT group treated with 3 days of successive 20 Gy fractions followed by 0.2 mg human IgG isotype i.p. 3 days after the final 20 Gy fraction, for a total of 5 doses; 5) Combination RT + HA PD-1 Ig, or 6) RT + mPD-L1 Ab groups treated with 3 days of successive 20 Gy fractions followed by i.p. injection with 0.2 mg HA PD-1 Ig, or mPD-L1 Ab, respectively, 3 days after the final 20 Gy fraction for a total of 5 doses.

### 2.14 Statistics

Data were analyzed using Prism 5 software (GraphPad Software Inc.). For the analysis of three or more groups, the non-parametric ANOVA, followed by post-test comparisons (Bonferroni for comparing all groups, or Dunnett's to compare each group to the control group), was used. Analysis of differences between two normally distributed groups was performed using the Student's *t*-test. Data were tested for normality and variance. A p value of < 0.05 was considered significant. All experiments were performed at least twice for reproducibility.

### 2.15 Study Approval

All animal studies were conducted according to protocols approved by the Institutional Animal Care and Use Committee (IACUC) of the Albert Einstein College of Medicine.

## 3 Results

### 3.1 Mapping the Ligand Binding Interface of Human PD-1

**Fig. 1:**
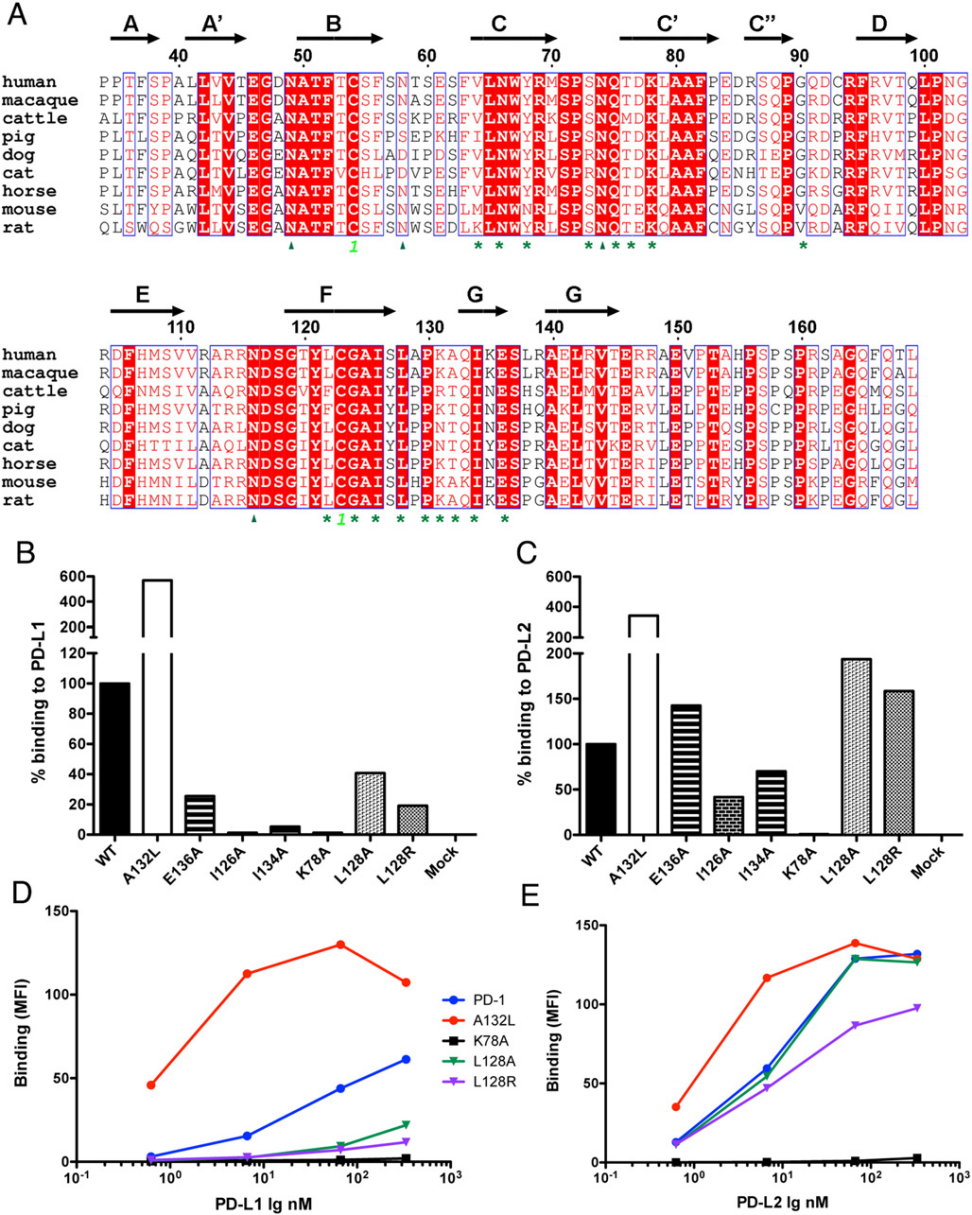
Mapping the ligand-binding surface of human PD-1 by site-directed mutagenesis. (A) Alignment of PD-1 sequences from various species. Structural annotations are based on the mouse PD-1 structure (pdb ID 1NPU). Asterisks indicate residues that contribute to binding PD-L1 and/or PD-L2. Glycosylation sites are denoted with triangles, cysteines forming disulfide bonds are marked with highlighted numbers. Relative binding of human PD-1 mutants to PD-L1 (B) and PD-L2 Ig (C), at 0.5 μg/ml ligand concentration, normalized to wild type PD-1 binding. Concentration dependent binding of selected human PD-1 mutants to (D) PD-L1 Ig and (E) PD-L2 Ig evaluated by flow cytometry. Representative data for 3 independent experiments are shown for B–E.

Based on our previously reported crystal structure of the mouse PD-1/PD-L2 complex (Lazar-Molnar et al., 2008b), and sequence similarity between PD-1 orthologs (Fig. 1A), mutants of human PD-1 were designed at positions contributing to the PD-1:PD-Ligand interface and expressed in HEK293 cells. Wild type and mutant PD-1 proteins showed similar expression levels, as detected by specific antibody staining (data not shown). Binding to PD-L1 and PD-L2 Ig fusion proteins was evaluated by a flow cytometry-based binding assay.

Consistent with earlier studies on mouse PD-1 (Lazar-Molnar et al., 2008b, Zhang et al., 2004), mutations of key residues on the front face of the human PD-1, such as the K78A mutation in the C′ strand, resulted in complete loss of binding to both PD-L1 and PD-L2 (Fig. 1B–C, Table 1). Another group of mutants, including I126A, I134A, L128A, L128R and E136A, exhibited reduced binding (40% or less) to PD-L1, but bound PD-L2 at levels similar to wild type, except I126A, which showed 50% lower binding to PD-L2 as well. Notably, the behavior of some PD-L2 selective mutants (i.e., I126A (F strand), I134A (G strand) and E136A) is specific for human PD-1, as analogous mutations in murine PD-1 results in loss of binding to both PD-Ligands (Lazar-Molnar et al., 2008b). Finally, a third group of mutants, including various changes of Ala132 to hydrophobic residues (e.g., Leu, Phe, Ile, Thr, Val) exhibit enhanced binding to both PD-L1 and PD-L2 (Fig. 1B–C, Table 1 and Supplementary Fig. 1). The binding behavior of selected mutants from each group was further confirmed by titration with various concentrations of PD-Ligands (Fig. 1D–E). The A132L mutant showed increased binding to both PD-L1 and PD-L2 at all concentrations tested, while no detectable binding was observed with K78A even at concentrations as high as 50 μg/ml. L128 mutants showed preferential binding to PD-L2, and no or reduced binding to PD-L1 (Fig. 1D–E).

The general similarity of the human PD-1 mutants presented here and the murine PD-1 mutants reported earlier is not surprising, as 14 of the 17 residues that contribute to the binding interfaces are conserved between mouse and human PD-1, with 9 residues being conserved in all species (Fig. 1A, Supplementary Fig. 2). These shared determinants represent the molecular basis of the substantial cross-reactivity observed between mouse and human PD-1 and PD-Ligands (Supplementary Fig. 3 and below).

### 3.2 Crystal Structure of the Human PD-1 A132L Mutant

**Fig. 2:**
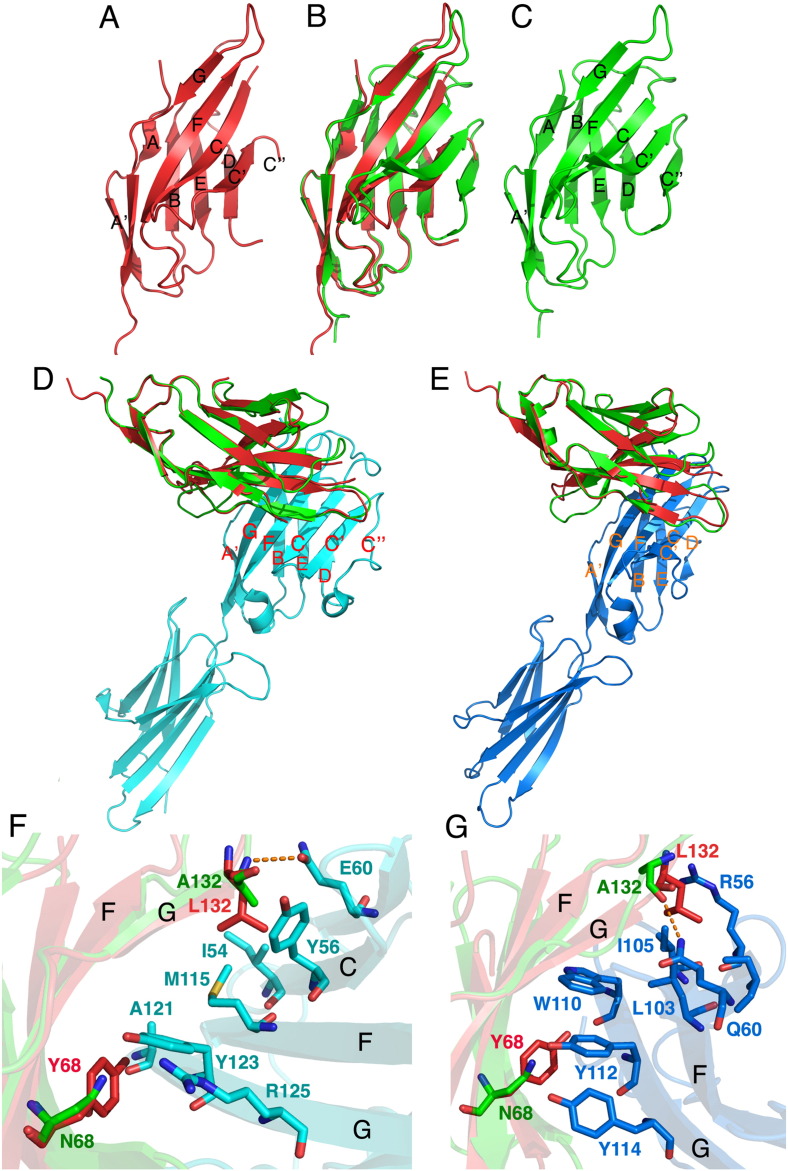
Crystal structure of human A132L mutant PD-1 shows similar overall architecture to mouse PD-1. (A) Human PD-1 IgV structure. (B) Superposition of human and mouse (RMSD 1.3 for 100 C_α_). (C) Mouse PD-1 IgV structure. (D) Superposition of human PD-1 structure with the mPD-1/huPD-L1 complex (pdb ID 3BIK), RMSD 1.1 Å for 100 Cα. (E) Superposition of the human PD-1 structure with mouse PD-1/PD-L2 complex (Pdb ID 3BP5), RMSD 1.1 for 98 Cα. (F) Model of the interface between human (A132L) and mouse PD-1 interacting with human PD-L1. (G) Details of the interactions of human (A132L) and mouse PD-1 with PD-L2. Red: human PD-1 A132L, green: mouse PD-1, cyan: human PD-L1, blue: mouse PD-L2. Dotted lines indicate hydrogen bonds.

To further examine the structural/biochemical basis for PD-1 function, we performed extensive crystallization trials using wild type, and A132L mutant human PD-1 refolded from inclusion bodies expressed in *E. coli*. Wild type PD-1 failed to yield diffraction quality crystals; however, the crystal structure of the high affinity A132L mutant of human PD-1 was successfully determined. The structure (residues N33 through A149) exhibits typical IgV domain topology, with the front β-sheet (strands A'GFCC'C″) and back β-sheet (strands ABED) forming a two layered β-sandwich stabilized by the canonical disulfide bond between the B (C54) and F (C123) β-strands (Fig. 2A). The human and murine (Zhang et al., 2004) PD-1 structures are similar (Fig. 2A–C), with an RMSD of 1.3 Å (over 100 C_α_ atoms), however, the presence of the C″ strand could not be modeled in the human PD-1 structure due to lack of electron density for that region. Subsequent to deposition of our structure, the solution NMR structure of wild type human PD-1 was reported, which exhibits a high degree of similarity, with an RMSD of 1.4 Å over 101 C_α_ atoms (Cheng et al., 2013). Consistent with the X-ray structure, the NMR structure exhibits extensive flexibility of the region between the C′ and D strands, with the absence of a well ordered C″ strand.

Superposition of the human A132L PD-1 structure with the structure of the mouse PD-1/human PD-L1 (3BIK) complex (Lin et al., 2008) shows little overall change of the receptor upon binding (RMSD 1.1 for 100 Cα) (Fig. 2D–E). However, this superposition highlights an important difference between the ligand binding surface of mouse and human PD-1, namely the presence of tyrosine 68 in the C strand, which is conserved in all species, with the exception of mouse PD-1, where it is replaced by asparagine (Fig. 1A). Y68 stacks against Y123 (G strand) in human PD-L1, the equivalent of which is conserved in all the known PD-L1 and PD-L2 sequences (Supplementary Fig. 2).

Superposition of the human PD-1 structure with the mouse PD-1/mouse PD-L2 complex (3BP5, RMSD 1.1 for 98 Cα) predicts that Y68 in human PD-1 contributes to a hydrophobic cluster including W110, Y112, and Y114 on the G strand of PD-L2 (Fig. 2E and G). PD-L1 lacks two of these aromatic residues, with W110 replaced by A121, and Y114 replaced by R125 in all the PD-L1 sequences; Y112 (123 in PD-L1) is conserved in all PD-L sequences and interacts with Tyr68 in human PD-1. The altered interactions associated with these substitutions may contribute to the higher affinity of human PD-1 towards PD-L2, compared to PD-L1.

The A132L mutation in human PD-1 is predicted to afford additional van der Waals interactions with Y56, I54 (C strand) and M115 (F strand) in human PD-L1 (Fig. 2F). Similarly, upon binding PD-L2, additional hydrophobic interactions could occur with L103 and I105 (F strand) of mouse PD-L2, and I103 and 105 of human PD-L2, respectively (Fig. 2G). We have been unable to produce diffraction quality crystals of the human A132L mutant in complex with human PD-L1, and we have not successfully refolded PD-L2, thus precluding crystallization trials with human PD-L2. However, we have determined the crystal structures of the complexes of the mouse analog of A132L with human PD-L1 (pdb ID 3SBW) and mouse PD-L2 (pdb ID 3RNK and 3BP6). Although there were no major differences in stoichiometry, or overall conformation between wild type and A132L PD-1 complexes, as predicted, the Leu residue present in the mutant made a greater number of interactions than the Ala residue in wild type PD-1 with PD-L1 and PD-L2, consistent with its higher affinity (data not shown).

### 3.3 Binding Affinities of Human Wild Type and High-Affinity A132L Mutant PD-1 With PD-L1 and PD-L2

The interactions of wild type, and A132L mutant PD-1 with PD-Ligands were quantified using Surface Plasmon Resonance (SPR) (Table 2). PD-L1 or PD-L2 Ig was immobilized to the surface of the sensor chip, and soluble monovalent PD-1 was injected. The binding processes for the human proteins are characterized by relatively fast k_on_ and k_off_ rates, similar to those observed earlier for the PD-1/PD-L interactions for mouse PD-1 (Lazar-Molnar et al., 2008b, Youngnak et al., 2003, Zhang et al., 2004). Fitting of the data to a 1:1 one-site binding model resulted in K_d-_s of 6.4 μM and 0.2 μM for the human PD-1:PD-L1 and PD-1:PD-L2 interactions, respectively (Table 2, Supplementary Fig. 3), revealing 30-fold higher affinity of human PD-1 to PD-L2, compared to PD-L1. In contrast, analysis of the murine orthologs yield K_d_s of 5.4 μM and 2.8 μM for the PD-1:PD-L1 and PD-1:PD-L2 interactions, respectively, (Table 2, Supplementary Fig. 4), resulting in ~ 2-fold difference in affinity between murine PD-L1 and PD-L2, which is consistent with earlier data (Youngnak et al., 2003, Zhang et al., 2004). In addition to its higher affinity for human PD-L2, human PD-1 was bound more strongly to mouse PD-L2, compared to mouse PD-1 (0.27 μM for human PD-1/mPD-L2, versus 2.8 μM for mouse PD-1/mPD-L2). Binding of human PD-1 to mouse PD-L1 was comparable to the mouse PD-1/PD-L1 interaction (3.8 μM for human PD-1/mPD-L1, versus 5.4 μM for mouse PD-1/mPD-L1) (Table 2, and Supplementary Fig. 4).

Analysis of the interaction of the human A132L mutant with human PD-L1 and PD-L2 yielded in K_d_ values of ~ 0.14 μM and ~ 6.5 nM, respectively. Thus, introduction of this single point mutation, A132L, into human PD-1 resulted in 45- and 30-fold enhanced affinities for human PD-L1 and PD-L2, respectively (Table 2). Compared to wild type murine PD-1, this human PD-1 mutant also exhibited enhanced affinities towards the murine PD-Ligands (K_d_ ~ 0.23 μM, 23-fold increased affinity for mPD-L1, and K_d_ ~ 0.029 μM, 96-fold increase for mPD-L2, Table 2), which supports the possibility for functional evaluation of this mutant in murine models.

### 3.4 Characterization of a High-Affinity Human PD-1 Ig Fusion Protein

Identification of the high affinity A132L PD-1 mutant motivated the design of a high-avidity bivalent dimeric PD-1 Ig fusion protein (HA PD-1 Ig). As a negative control, we generated K78A mutant human PD-1 as an Ig fusion, as it does not bind to either PD-L1 or PD-L2 (*i.e.*, non-binding, or NB PD-1 Ig). These two mutants, as well as wild type human PD-1, were expressed in HEK293 cells as soluble Ig fusion proteins linked to the Fc fragment of human IgG1 (Supplementary Fig. 5A), and purified from the supernatants of stably transfected cells by affinity chromatography. The purified Ig fusion proteins were examined for binding to PD-L1 and PD-L2 expressed on the surface of HEK293 cells using a flow-cytometry based assay. Consistent with the SPR binding experiments, the human A132L mutant exhibited increased binding to human PD-L1 and PD-L2, compared to the wild type PD-1, while no significant binding was detected between K78A PD-1 Ig and either PD-L1 or PD-L2 (Supplementary Fig. 5B–C). HA PD-1 Ig also showed enhanced binding to mouse PD-L1 and PD-L2 (Supplementary Fig. 5D–E), supporting the feasibility of its use to selectively manipulate the PD-1/PD-Ligand pathways *in vitro* and *in vivo*.

### 3.5 High-Affinity PD-1 Ig Exhibits Increased Binding to Human Monocyte-Derived Dendritic Cells

**Fig. 3:**
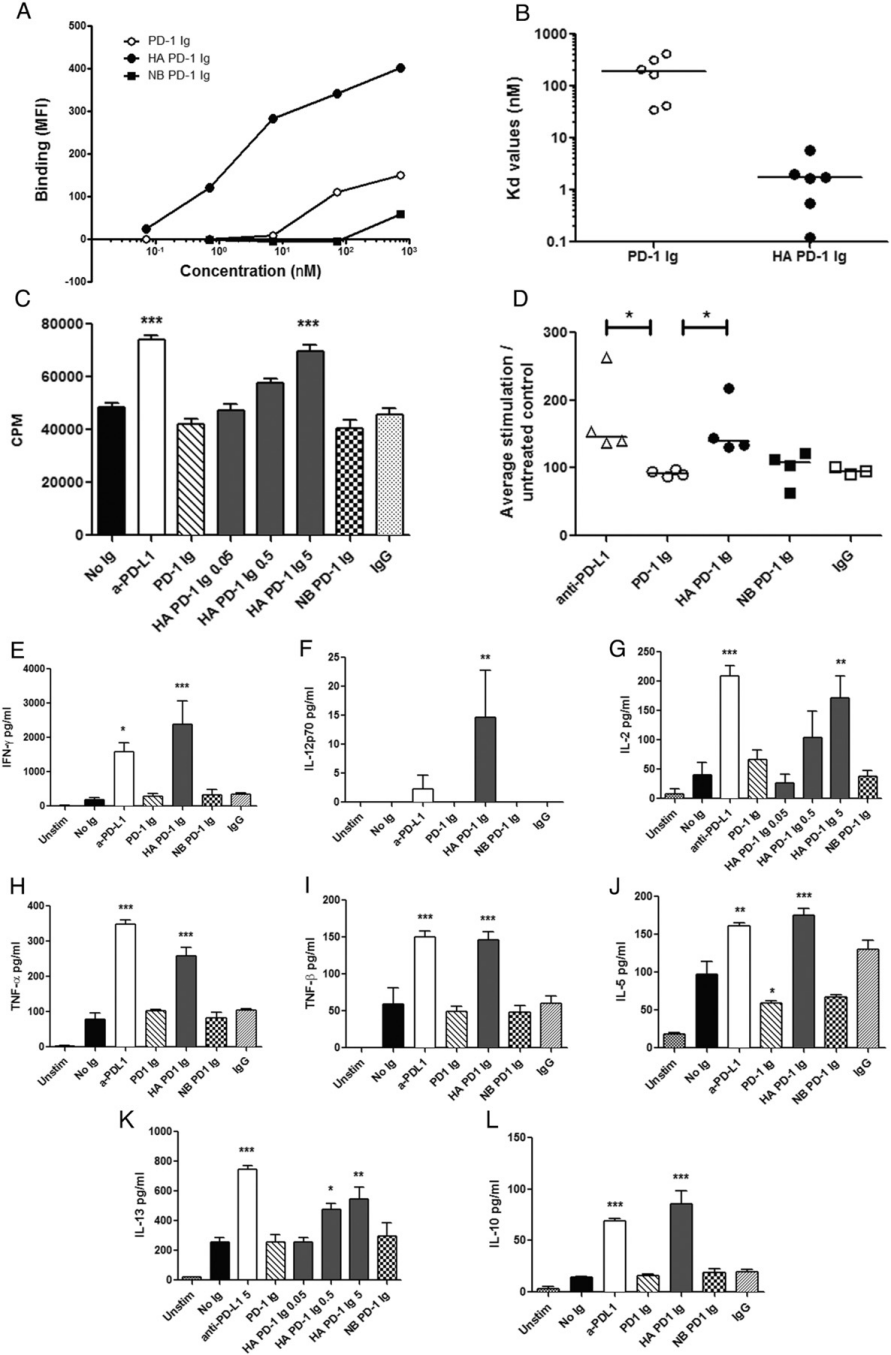
High-affinity A132L PD-1 Ig increases T cell proliferation and cytokine production *in vitro* after allogeneic stimulation. (A) A132L PD-1 Ig shows increased binding to human mature dendritic cells expressing PD-L1 and PD-L2, compared to wild type PD-1. Non-binding (NB) PD-1 Ig shows negligible binding only at high concentration (50 μg/ml). Data are representative of five independent experiments. (B) K_d_ values calculated from binding data for wild type and HA PD-1 Ig, respectively; the horizontal lines indicate the median values. K_d_-s were obtained by one-site fitting of the binding curves (Y = Bmax ∗ X/(K_d_ + X), where X is the concentration in nM, Y is specific binding expressed as MFI; Bmax: maximum binding). Data from five independent experiments are shown, analyzed by non-parametric Wilcoxon rank test, *p < 0.05. (C) Increased CD4^+^ T cell proliferation measured in the presence of HA PD-1 Ig, but not wild type PD-1 Ig, relative to the untreated group (no Ig). Blocking antibody to PD-L1 was used as positive control, K78A (NB) mutant PD-1 Ig or human IgG were used as negative controls. Groups were compared by ANOVA, followed by Bonferroni's post-test, ***p < 0.0001. (D) T cell proliferation is enhanced by anti-PD-L1, and HA PD-1 Ig, but not wild type PD-1 Ig, in allogeneic MLR reactions. Proliferation was normalized to the untreated control (100%) within each experiment. Data from 4 independent experiments are shown, each symbol representing average normalized value (of 4 replicates) from one individual experiment. Horizontal bars represent median values. Treatments were compared to wild type PD-1 Ig, (Kruskall-Wallis test, *p < 0.05). Secretion of T cell cytokines IFN-γ (E), IL-12 (F), IL-2 (G), TNF-α (H), TNF-β (I), IL-5 (J), IL-13 (K), and IL-10 (L) is enhanced after PD-1 blockade in the presence of either HA PD-1 Ig, or PD-L1 blocking antibody. Treatment reagents were used at 5 μg/ml, unless noted otherwise on the graphs. Groups were compared by ANOVA, followed by Dunnett's post-test, ****p < 0.0001, ***p < 0.001, **p < 0.01, *p < 0.05.

To evaluate the efficacy of our soluble mutant PD-1 Ig reagents in modulating the PD-1/PD-Ligand pathways, we first measured their binding to dendritic cells differentiated *in vitro* from monocytes of healthy donors in the presence of cytokines (GM-CSF and IL-4). After treatment with TNF-α to induce a ‘mature’ phenotype, these dendritic cells expressed both PD-L1 and PD-L2 (Supplementary Fig. 6). Notably, the HA PD-1 Ig exhibited enhanced binding, determined by flow cytometry, compared to wild type PD-1 Ig (Fig. 3A), with an average apparent K_d_ of ~ 2 nM (Fig. 3B), equivalent to ~ 100-fold greater avidity than that of wild type PD-1.

### 3.6 High-Affinity PD-1 Ig is a Potent Blocker of the PD-1/PD-Ligand Pathway in an Allogeneic MLR

The *in vitro* functional activity of HA PD-1 Ig was examined using an allogeneic mixed lymphocyte reaction (MLR) assay as a model for T cell activation. CD4^+^ T cells purified from the peripheral blood of healthy donors were co-cultured for 5 days with allogeneic monocyte-derived mature dendritic cells pre-treated with the Ig fusion proteins, and subsequent T cell proliferation was measured by ^3^H-thymidine uptake. HA PD-1 Ig elicited a dose dependent enhanced of T cell proliferation, with observable effects at concentrations as low as 0.5 μg/ml, and a significant increase at 5 μg/ml (Fig. 3C). In contrast, wild type PD-1 Ig had no significant effect at concentrations as high as 50 μg/ml (data not shown). The effect of HA PD-1 Ig at 5 μg/ml was comparable to that elicited by the same concentration of function blocking monoclonal antibody to PD-L1 (clone MIH1, Fig. 3C–D). No stimulation was observed with the non-binding K78A mutant PD-1 Ig used as negative control.

Along with increasing lymphocyte proliferation, HA PD-1 Ig elicited significant dose dependent enhancements of activation-induced cytokine production in the T cell-dendritic cell co-cultures (Fig. 3E–L). Increased levels of Th1 type cytokines IFN-γ, IL-12p70, IL-2 and Th2 type cytokines IL-5 and IL-13 were detected in the HA PD-1 Ig treated wells; secretion of the pro-inflammatory cytokines TNF-α and TNF-β, and the anti-inflammatory cytokine IL-10 was also enhanced (Fig. 3G and K and Supplementary Fig. 7). Overall, the effect of HA PD-1 Ig on cytokine secretion was comparable to that of a function blocking anti-PD-L1 antibody. Although Th1 cytokines IFN-γ and IL-12 p70 showed a trend towards more prominent increase after HA PD-1 Ig treatment, compared to anti-PD-L1 (Fig. 3E–F), these differences were not statistically significant.

### 3.7 Combined Radiation Treatment and HA PD-1 Ig Administration Synergistically Inhibit Primary and Metastatic Tumor Growth, and Promote Memory Anti-Tumor Responses

**Fig. 4:**
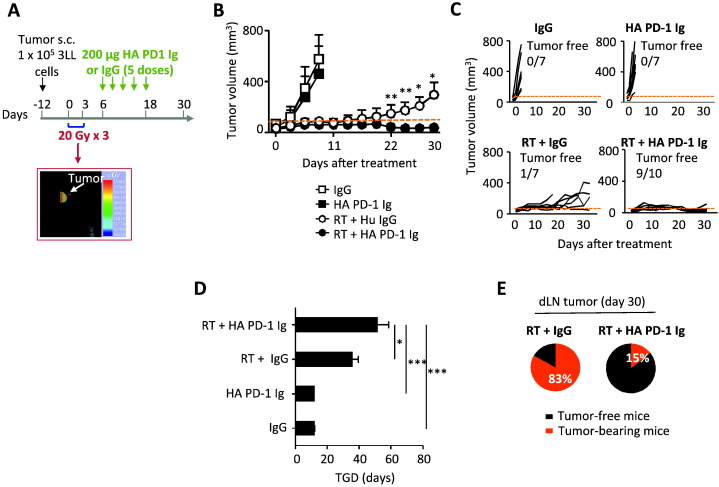
The combination of HA PD-1 Ig and hypofractionated radiation therapy (RT) results in synergistic control of Lewis lung carcinoma in mice. (A) Treatment plan: C57BL/6 mice were injected subcutaneously with 10^5^ Lewis lung carcinoma (3LL) cells (black arrow). Mice with a tumor volume of ~ 50 mm^3^ were randomized into 4 groups (IgG, HA PD-1 Ig, RT + IgG, and RT + HA PD-1 Ig) and received either a total of 60 Gy radiation, administered in 3 sequential 20 Gy fractions, or i.p. injections of 200 μg HA PD-1 Ig or isotype control (IgG) every 3 days for a total of 5 doses starting on day 6, as indicated by green arrows. The insert (red arrow) shows a footpad tumor visualized by on-board cone-beam computed tomography (CBCT). (B and C) Measurements of cumulative and individual tumor volumes. Data are representative of 3 independent experiments (n = 7–9). The horizontal dotted line indicates the treatment starting tumor volume (~ 50 mm^3^). (D) Tumor growth delay (TGD). (E) Percentage of treated mice in RT + IgG and RT + HA PD-1 Ig groups that developed a secondary tumor in the draining lymph node (dLN) 30 days post-treatment. Bars on graphs show mean ± SD. *p < 0.05, **p < 0.01, ***p < 0.001 (Two-way ANOVA or Student's *t*-test).

To investigate the *in vivo* efficacy of HA PD-1 Ig for immunotherapy, we used a Lewis lung carcinoma (3LL) model (Chakravarty et al., 1999, Chakravarty et al., 2006, Deb et al., 2001) to evaluate the synergy between HA PD-1 Ig-mediated checkpoint inhibition and radiotherapy (RT) (Fig. 4A). Tumors were induced in C57BL/6 mice by subcutaneous injection of 1 × 10^5^ 3LL cells in the dorsum of the right hind limb. About 12 days after tumor inoculation, when tumors reached ~ 50 mm^3^, mice were randomized into 4 treatment groups: HA PD-1 Ig + RT, HA PD-1 Ig, human control IgG (IgG), and RT + IgG. Two groups, RT + IgG and HA PD-1 Ig + RT, received three consecutive fractions of radiation (20 Gy) specifically targeting the tumor, starting on day 1 (about 12 days after tumor cell inoculation), using a hypofractionated image-guided radiation therapy (IGRT) (Bandyopadhyay et al., 2016) operated on a small animal radiation research platform (SARRP) equipped with on-board cone beam computed tomography (CBCT) (Brodin et al., 2015) (Fig. 4A). HA PD-1 Ig or control IgG were injected intraperitoneally (0.2 mg/mouse) in the appropriate groups starting on day 6 (with respect to completion of last RT treatment) with 5 doses at 3-day intervals, based on prior work using PD-1 blocking antibody (Lazar-Molnar et al., 2008a). As expected, both the IgG and the HA PD-1 Ig groups failed to control tumor growth, requiring sacrifice of animals as early as day 9 (Fig. 4B and C), while both RT + IgG and RT + HA PD-1 Ig groups showed low stable tumor volumes from days 9–20. However, after day 20, the RT + IgG treated group showed significant tumor growth, while the RT + HA PD-1 Ig treated group exhibited continued control of tumor growth extending to day 30 (the last day of observation) (Fig. 4B and C). While none of the mice in the IgG and the HA PD-1 Ig groups, and only 1 out 7 mice in RT + IgG group, achieved tumor-free status, in the RT + HA PD-1 Ig group 9 out 10 mice were tumor free (Fig. 4C). The RT + HA PD-1 Ig group showed a significant tumor growth delay (TGD) as compared to RT + IgG and the control groups (IgG or HA PD-1 Ig) (Fig. 4D), and a significantly lower tendency to develop secondary tumors (15% *vs* 83% in RT + HA PD-1 Ig and RT + IgG mice, respectively) in the distal inguinal LN (Fig. 4E) at 30 days post-treatment. These findings demonstrate that localized radiation treatment combined with HA PD-1 Ig induces a synergistic anti-tumoral effect that results in a more efficient tumor control than either of the individual treatments.

**Fig. 5:**
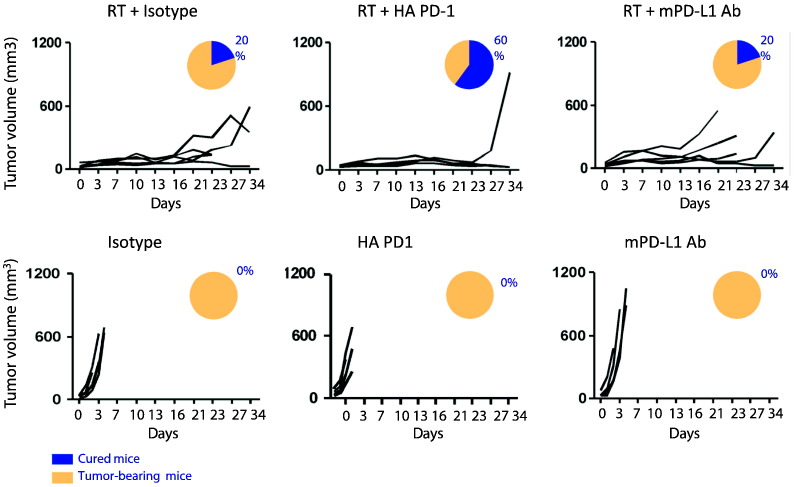
Superior synergistic effect of HA PD-1 Ig combined with hypofractionated radiation therapy (RT) in inducing tumor regression. Tumor cells were subcutaneously transplanted into C57BL/6 mice. Groups of randomized mice (5 mice/group) were treated i.p. with 200 μg HA-PD1 Ig or PD-L1 blocking antibody either alone, or in combination with three doses of 20Gy as described for Fig. 4. For controls, mice were injected with 200 μg of IgG2a or IgG1. Tumor growth was monitored and is shown as volume (mm^3^). Proportion of cured (blue) versus tumor-bearing (yellow) mice is indicated for each treatment.

To further characterize the anti-tumor effect of our HA PD-1 Ig we compared it to a function blocking anti-PD-L1 monoclonal antibody (mPD-L1 Ab clone 10F.9G2), in combination with radiation therapy using the same 3LL lung tumor model. We found that the synergistic effect of HA PD-1 Ig with radiation therapy in inducing tumor regression was superior to that observed with mPD-L1 Ab, or radiation therapy alone (Fig. 5, top panels). Our data indicate that 60% of the mice treated with HA PD-1 Ig combined with RT were tumor free in contrast to only 20% of mice treated with mPD-L1 mAb and radiation. HA PD-1 Ig or anti-PD-L1 alone had no effect on tumor growth in this model, showing no difference compared to the isotype control (Fig. 5, lower panels).

**Fig. 6:**
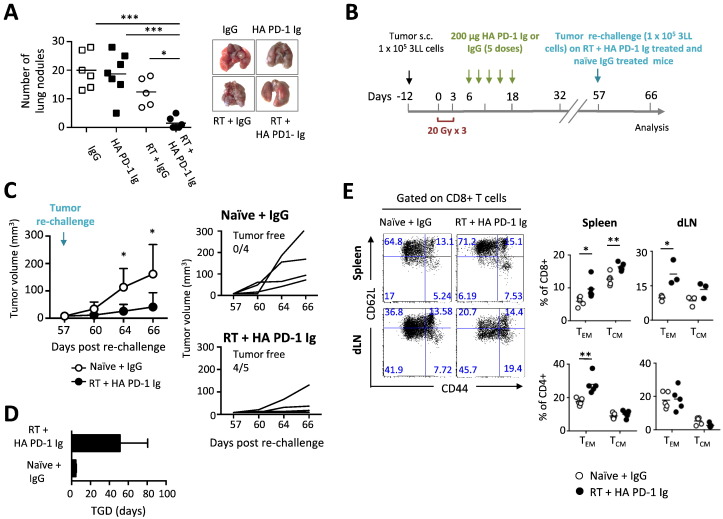
Combination of HA PD-1 Ig and hypofractionated radiation therapy (RT) reduces lung metastatic burden and promotes immunological memory responses. (A) Quantitation and visualization of metastatic lung nodules in treated mice that received below-the-knee amputation (B) Treatment plan to study memory response: C57BL/6 mice were injected subcutaneously with 10^5^ Lewis lung carcinoma (3LL) cells (black arrow) and treated as previously described. Mice that were “cured” were re-challenged at day 57 with 1 × 10^5^ 3LL cells in the left foot, monitored and sacrificed and day 66 for memory responses study. As a control for tumorigenicity, naïve control mice were inoculated with the same number of 3LL cells. (C) Measurement of cumulative and individual tumor volumes in naïve and RT + HA PD-1 Ig mouse groups. Tumor volumes were recorded 2–3 times per week. Data are representative of two independent experiments (n = 4–5/group); mean ± SD is shown. (D) Tumor growth delay (TGD). (E) Representative flow cytometry dot plots of effector memory (T_EM_, CD44^hi^CD62L^lo^) and central memory (T_CM_, CD44^hi^CD62L^hi^) CD8 and CD4 T cells from spleen and dLN. Numbers in each quadrant indicate the relative percentage of population. Adjacent scatter plots represent the frequency of memory subsets reported per mouse. Bracketed lines indicate means. Data are representative of 2 independent experiments (n = 3–5/group). *p < 0.05, **p < 0.01 (Student's *t*-test).

Since the Lewis carcinoma cell line is highly metastatic, the efficacy of the combination treatment to control the development of lung metastases was examined. Mice treated with single therapies (control IgG, HA PD-1 Ig and RT), followed by amputation of the tumor bearing foot (approximately 21 days after tumor inoculation) began exhibiting high numbers of pulmonary metastatic nodules at 3–4 weeks post-treatment. The mean metastatic colony counts were not significantly different between the control, HA PD-1 Ig, and RT + IgG groups (20.0 ± 2.6, 18.7 ± 2.9, and 12.4 ± 2.2, respectively). In contrast, the number of metastatic nodules in the RT + HA PD-1 Ig group (1.5 ± 0.58) was significantly reduced, as evidenced by images of the lungs (Fig. 6A).

Finally, we assessed whether the combination therapy could induce an immunological memory. “Cured” mice from the RT + HA PD-1 Ig group, were re-challenged at day 57 with the same number of tumor cells (1 × 10^5^ 3LL cells) in the opposite foot and monitored for tumor growth (Fig. 6B). Naïve tumor-bearing mice were used as control and injected with control IgG. Our findings revealed significant tumor regression (Fig. 6C) in RT + HA PD-1 Ig group as compared to control naïve mice and a significantly higher TGD (Fig. 6D). These results correlated with a significant enrichment of T effector memory cells (T_EM_) in both CD4^+^ and CD8^+^ T cells in the spleen and draining lymph node (dLN), and by an accumulation of CD8^+^ T central memory cells (T_CM_) in the spleen (Fig. 6E). However, no changes were observed in the pool of CD4 central memory cells in the spleen or draining lymph node (Fig. 6E).

These *in vivo* experiments demonstrate that HA PD-1 Ig in combination with radiation therapy synergistically enhances control of local and metastatic tumor burden, and promotes immunological memory responses, which afford significant protective anti-tumoral immunity.

## 4 Discussion

The PD-1/PD-Ligand pathways are major regulators of effector T cell responses in normal and pathologic conditions, including cancer, infectious and autoimmune disease, and have emerged as important targets for cancer immunotherapy (Sharma and Allison, 2015a, Sharma and Allison, 2015b). Herein, we report the crystal structure and detailed mutagenesis analysis of human PD-1, which resulted in the identification of a high-affinity human PD-1 mutant. Based on this finding, we generated a mutant PD-1 Ig fusion protein with enhanced avidity for both human PD-L1 and PD-L2. The cross-reactivity of this bivalent high-affinity reagent with the murine PD-Ligands enabled demonstration of *in vitro* and *in vivo* activities consistent with a candidate immunotherapy that can elicit blockade of both PD-L1 and PD-L2-mediated processes.

Structure guided mutagenesis of human PD-1 resulted in several classes of mutants (Table 1). The first class of mutants (K78A and I126A) exhibited reduced or no binding to both ligands. Only the K78A mutant resulted in undetectable binding to both PD-L1 and PD-L2; while the I126A mutant showed no binding to PD-L1 and ~ 50% reduced binding to PD-L2.

A second class of mutants (I134A, L128A, L128R and E136A) exhibited close to wild type affinities towards human PD-L2, but significantly reduced affinities towards human PD-L1. These PD-L2 specific mutants could potentially be used to selectively study the role of PD-L1 and PD-L2 in regulating the immune response. A third important class of human PD-1 mutants exhibited increased affinity to both human PD-L1 and PD-L2. In particular, mutation of alanine 132 to leucine, or other hydrophobic residues such as Ile, Phe, Val or Thr, resulted in enhanced binding to both ligands (Supplementary Fig. 1), as observed in cell-surface binding experiments using HEK293 transfected cells.

A naturally occurring A132V variant, similar to our high-affinity mutant, has been reported (ExAC database, http://exac.broadinstitute.org/ (Lek et al., 2016)), and is a confirmed somatic mutation in colon cancer (Cosmic database, cancer.sanger.ac.uk (Forbes et al., 2015)). This substitution has been reported as neutral or benign, although in the light of our data it is expected that the A132V substitution would result in higher affinity to both PD-L1 and PD-L2 (Supplementary Fig. 1). Additional naturally occurring variants include E136Q, which has been detected in cervical cancer and reported as probably damaging. Although we have not evaluated the E136Q mutant, the E136A mutant showed loss of binding to PD-L1 (Fig. 1B–C), suggesting that this position is important for PD-1 function (Fig. 1B–C).

While the human and mouse PD-1 mutants tend to show generally similar behavior in terms of ligand binding, including the high-affinity A132 mutants, there are some important differences. In contrast to murine PD-1, where mutations of residues I134 and E136 result in complete loss of binding to both ligands (Lazar-Molnar et al., 2008b), analogous mutations in human PD-1 result in loss of binding only to PD-L1. This observation supports the notion that human PD-1 binds more strongly to PD-L2, compared to mouse PD-1. Surface plasmon resonance (SPR, Biacore) experiments confirmed that the human PD-1/PD-L2 interaction was 15-fold stronger than the mouse PD-1/PD-L2 interaction, and human PD-1 bound to mouse PD-L2 with 10-fold higher affinity, compared to mouse PD-1 (Table 2).

Notably, in contrast to the modest 2-fold difference between the mouse PD-1/PD-L2 versus PD-1/PD-L1 interaction, human PD-1 showed 30-fold stronger binding to PD-L2, compared to PD-L1 (K_d_ 0.2 versus 6 μM). A recent study by Cheng et al. reported similar K_d_ value for the human PD-1/PD-L1 interaction (8.2 μM), but only ~ 4-fold stronger affinity for the PD-1/PD-L2 (2.3 μM) interaction (Cheng et al., 2013). This difference could be due to modest differences in experimental conditions such as the detailed features of the expression construct and the conditions used for the binding experiments (coupling system, temperature).

Similar binding experiments performed with the A132L mutant human PD-1 showed over 45, and 30 fold increased binding to PD-L1, and PD-L2, respectively, compared to the wild type, which was largely due to a slower dissociation rate. Although our previous mutagenesis studies on mouse demonstrated that the mouse A132L mutant exhibited enhanced affinity for mouse PD-L1 and PD-L2 (Lazar-Molnar et al., 2008b, Zhang et al., 2004), the increase in affinity for the mouse mutant was only 2-, and 3-fold, respectively, which is at least one order of magnitude lower, compared to the human counterparts.

The human high-affinity mutant A132L selected for further studies showed enhanced binding not only to human PD-L1 (45 fold) and PD-L2 (30 fold), but also to the corresponding mouse ligands. Binding of the human A132L mutant to mouse PD-L1 was enhanced 23 fold (Kd ~ 0.23 μM), compared to the endogenous mouse PD-1/PD-L1 (Kd ~ 5.4 μM) interaction, and binding to mouse PD-L2 was increased 96 fold (Kd ~ 29 nM), compared to the endogenous murine PD-1/PD-L2 interaction (Kd ~ 2.8 μM). Due to this binding behavior, the A132L high-affinity human PD-1 (HA PD-1) is suitable for use in *in vivo* mouse models, consistent with the results reported herein. Moreover, it is expected that HA PD-1 would recognize non-human primate PD-L1 and PD-L2 with affinities and selectivities comparable to the human ligands, since the residues involved in the binding interfaces are strictly conserved. Overall there is ~ 99% identity between human and chimpanzee PD-L1 (one residue is different in the cytoplasmic domain, R260C change) and 95.1% identity between human and chimpanzee PD-L2 proteins (human sequence 10 residue shorter in the cytoplasmic tail, and three residues differ: R70C in C′ strand and S86L in E strand, not part of binding interface; F229S in transmembrane domain). By comparison, the human and mouse PD-Ligands exhibit ~ 70% identity only. The cross reactivity of the HA PD-1 reagent is a significant feature, which precludes the need to re-engineer the biologic when moving between experimental systems, in contrast to monoclonal antibodies which are typically species-specific.

Determination of the human PD-1 (A132L mutant) crystal structure allowed for the detailed analysis of the residues contributing to the ligand-binding interfaces. Human PD-1 exhibits a monomeric IgV domain structure similar to the mouse PD-1 crystal structure (pdb ID 1NPU, (Zhang et al., 2004)). Superposition of human PD-1 structure with the mouse PD-1/hu PD-L1 (RMSD 1.1 Å for 100C) and mouse PD-1/PD-L2 (RMSD 1.1 for 98 Cα) complexes showed overall similar arrangements. The most significant difference between human and mouse PD-1 is the absence of the C″ strand in the human structure, which is consistent with the subsequently reported NMR structure (Cheng et al., 2013). Another important difference is the presence of a tyrosine in the ligand-binding site of human PD-1 at position 68, which is not conserved in mouse PD-1. Tyr68 in human PD-1 appears to contribute to the higher affinity to PD-L2 due to enhanced van der Waals contacts with a hydrophobic cluster of residues in PD-L2 (W110, Y112 and Y114) in the human PD-1/PD-L2 complex. These interacting residues are highly conserved in PD-L2 orthologs, with only 4 out of the 14 residues contributing to the PD-1 recognition surface differing between mouse and human PD-L2 (positions 56, 101, 103 and 108, Supplementary Fig. 2). Although the structure of the human PD-1/PD-L2 complex has not been reported, based on existing structures, it is likely that some of these residues could also influence the strength of the PD-1/PD-L2 interaction (e.g. presence of Val in human PD-1 versus Ala in mouse at position 108; or Thr in human versus Arg in mouse at position 56). In contrast to human PD-1, the asparagine side chain at position 68 in mouse PD-1 is unable to form hydrophobic interactions with the aromatic residues from PD-L2, although it forms a hydrogen bond with the OH group of Tyr112. Interestingly, with the exception of mouse PD-1, Tyr68 is conserved in all the known PD-1 sequences, which suggests that it could have arisen as an evolutionary gain-of-function mutation, resulting in increased affinity of PD-1 for PD-L2.

The human PD-1 mutant (A132L) exhibiting increased affinity for both PD-L1 (45-fold) and PD-L2 (30-fold) was selected for further studies and the generation of a soluble chimeric Ig fusion protein (HA PD-1 Ig). As expected, compared to wild type PD-1 Ig, the HA PD-1 Ig showed significantly enhanced binding to mature human dendritic cells expressing PD-L1 and PD-L2. Although it is not feasible to define a quantitative K_d_ in this cell-based system, due to a number of factors (e.g. variations in PD-Ligand expression levels, bivalency of the Ig fusion proteins, and this is not an equilibrium experiment), appreciation of the binding responses (Fig. 3A–B) indicates that the HA PD-1 Ig binds with an apparent affinity at least two orders of magnitude greater than the wild type PD-1 Ig. These data suggest that the apparent cell-surface binding of the HA PD-1 Ig is in the low single digit nanomolar range (~ 2 nM), similar to many antigen antibody complexes.

The enhanced binding of HA PD-1 Ig was consistent with the results of an allogeneic *in vitro* T cell activation assay, in which the HA PD-1 Ig increased T cell proliferation by at least 50%, (Fig. 3C–D), and the production of the Th1 cytokines IFN-γ and IL-12p70 6-fold or more; IL-2, IL-10, TNF-α, IL-13 by 4–5 fold; and IL-5 and TNF-β 2–3 fold, compared to untreated control (no Ig). The increase in proliferation and cytokine secretion for HA PD-1 Ig was dose-dependent, starting at concentrations as low as 0.5 μg/ml (Supplementary Fig. 7). Wild type PD-1 Ig did not induce significant T cell proliferation or cytokine production, except at concentrations in the 50 μg/ml range, which is above the usual concentration for such *in vitro* assays.

The effects of HA PD-1 Ig on proliferation and cytokine production suggests two possible mechanisms: the first involving competitive binding, which sterically blocks the endogenous cell surface resident ligands from engaging cell surface resident PD-1; and the second exploiting reverse signaling through PD-L1 and PD-L2 on APCs elicited by the soluble high-affinity PD-1. A recent study indicates that PD-L1 may signal in tumor cells, such as ovarian cancer and melanoma, through a cancer cell-intrinsic, non-immune mechanism (Clark et al., 2016). No such data are available for PD-L2, which has not been extensively investigated in lung tumor models. Relevant to the current study, 3LL cells express PD-L1 *in vitro*, but not PD-L2 (Supplementary Fig. 8). Flow cytometric analysis of tumor-associated (Supplementary Fig. 9A) and splenic DCs (not shown) did not show significant increase in surface activation markers after treatment, although there was a trend towards increased expression of PD-L1 (Supplementary Fig. 9B), likely induced by IFN-γ release following radiation therapy. Furthermore, the allogeneic MLR studies utilized irradiated DCs, indicating that the enhanced proliferation and cytokine production elicited by HA PD-1 Ig (Fig. 3.) was due to direct inhibitory effects on T cells and not through reverse signaling into the APCs. Importantly, the effects of the HA PD-1 Ig are comparable to that of a PD-L1 blocking monoclonal antibody used at the same concentration. Based on these observations, the *in vitro* behavior of our high affinity mutant PD-1 Ig suggests it possesses utility as a modulator of the PD-L/PD-1 pathway, with a potency that is in the range of functionally blocking antibodies.

Recent clinical trials highlighted the induction of anti-tumor immunity against various solid tumors by blockade of T cell inhibition through PD-1 and PD-L1 (Brahmer et al., 2012). These treatments resulted in durable tumor response rates of 10–15%, the highest rate for any immunotherapy approach for the treatment of cancer in the last 30 years (Ribas, 2012). Moreover, in addition to metastatic melanoma, promising results were obtained in patients with non-small cell lung cancer, which has been largely resistant to immunotherapy. Phase 3 clinical trials have recently shown unprecedented successes for the treatment of NSCLC using anti-PD-1 monotherapy compared to platinum-based chemotherapy (Killock, 2016). It is clear that not all patients respond to these treatments, and accumulating evidence indicates that tumors that are immunogenic may be more responsive to this form of immunotherapy. Hence, there exists a continued need to examine new immunomodulatory agents and explore new combination therapies that result in synergistic responses between immunotherapy and other modalities such as chemotherapy and radiotherapy (Shahabi et al., 2015).

Radiation therapy (RT) induces tumor cell death by eliciting irreparable DNA damage and cell cycle arrest, thus providing a robust source of antigens. By upregulating MHC class I expression, RT enhances antigen presentation and T cell recognition of irradiated tumor cells, sensitizing them to cytotoxic T cell-mediated clearance. RT further affects the tumor microenvironment by inducing increased expression of pro-inflammatory cytokines, such as TNF-α, IFN-γ, IL-1β, IL-6 and IL-12, type-I interferons and chemokines, which stimulate infiltration of immune cells into the tumor (Haikerwal et al., 2015). Recent technological advances, such as image-guided radiation therapy (IGRT) using cone beam computed tomography (CBCT), allow for great accuracy in delivering radiation to the targeted tumor volume, while preserving the surrounding healthy tissues. We examined the efficacy of our HA PD-1 Ig for immunotherapy using a Lewis lung carcinoma model and evaluated combined checkpoint inhibition and localized radiotherapy. Our *in vivo* experiments demonstrate that immune checkpoint inhibition, using HA PD-1 Ig, in combination with RT significantly enhances the control of primary and metastatic tumor growth. Morerover, the synergistic effect of HA PD-1 Ig/RT combination therapy on controlling tumor growth was more efficient than treatment with a monoclonal PD-L1 blocking antibody, or its combination with RT. These findings indicate that HA PD-1 Ig may be an important and complementary addition to the currently available monoclonal antibodies targeting PD-1 and PD-L1, and further suggest that relevant clinical trial studies may be a promising path. Analogously, biologics targeting the TNF pathway include both monoclonal antibodies to TNF (infliximab, adalimumab), and TNF receptor Ig fusion proteins (etanercept), each with their specific pharmacological profile.

The 3LL cells used in our murine model expresses PD-L1 (Supplementary Fig. 8), and radiation-induced pro-inflammatory cytokines such as IFN-γ have the potential to further upregulate PD-L1 expression in the tumor microenvironment, which could contribute to immune evasion by inhibiting PD-1-expressing effector T cells. Blockade of the PD-L1/PD-1 pathway offers the potential to reverse this immunosuppression, and increase the efficiency of T cell-mediated anti-tumor response.

In addition to reducing the size of primary footpad tumors and substantially increasing tumor growth delay, the combination HA PD-1 Ig + RT treatment resulted in a significant reduction of tumor metastasis to the local lymph nodes, as well as distant metastasis to the lungs. The lungs isolated from the combination HA PD-1 Ig and RT group contained very few nodules, compared to all the other groups, which showed extensive metastatic lesions over the entire surface of the lung lobes. Quantification of the metastatic lesions in the lungs showed dramatically reduced metastatic burden in RT + HA PD-1 Ig combination treatment group compared to all the other treatment groups. RT therapy alone slightly reduced the number of metastatic nodules in the lung, but this decrease was not significantly different from the control or HA PD-1 Ig single groups. Interestingly, only the combination radio-immunotherapy resulted in significantly reduced local and distal tumor metastasis in this Lewis lung carcinoma model, consistent with the desired synergistic effect.

Finally, the HA PD-1 Ig and RT combination therapy promoted a protective immunological memory response characterized by an accumulation of CD8 effector memory T cells (T_EM_), and CD8 central memory T cells (T_CM_) in “cured” mice that were re-challenged with a second injection of tumor cells 57 days after the initial tumor challenge. The primary challenge data showed an increase in tumor infiltrating lymphocytes (TILs) at about day 12 in the HA PD-1 Ig + RT combination group (Supplementary Fig. 9C), while at later time points no significant influx of TILs was observed (data not shown). Since memory T cells have less stringent requirements for activation than naïve cells e.g., responsiveness to lower concentrations of antigen, (Curtsinger et al., 1998, Pihlgren et al., 1996, Rogers et al., 2000), we hypothesized that memory CD8 T cells were more efficiently activated during tumor re-challenge, resulting in accumulation in the spleen as early as day 66 (9 days after re-challenge). This observation is consistent with the finding that only 15% of the mice in the RT + HA PD-1 Ig group developed local metastasis in the dLN, as opposed to over 80% of the RT group. Thus, our data suggest that combined radio-immunotherapy promotes the development of anti-tumor immunity capable of protecting against tumor recurrence.

Our results are in accordance with recent studies using RT combined with mAb-mediated blockade of PD-1 or PD-L1 in mouse models of melanoma (Sharabi et al., 2015), breast carcinoma (Deng et al., 2014, Sharabi et al., 2015), colon adenocarcinoma (Deng et al., 2014), glioma (Zeng et al., 2013) and multiple myeloma (Jing et al., 2015). Tumor bearing PD-1 deficient mice treated with stereotactic ablative radiotherapy showed better survival than wild type mice in preclinical melanoma and renal cell carcinoma models, and PD-1 blockade in wild type mice recapitulated the anti-tumor effect observed in PD-1 deficient mice (Park et al., 2015). Our study extends these observations to include a model of metastatic lung cancer. These studies demonstrate that PD-1 blockade potentiates the anti-tumor immune response induced by radiation therapy (RT), including significant effects on non-irradiated or secondary tumors, suggesting that this combination therapy may be a promising therapeutic approach for managing metastatic cancer patients.

Multiple mechanisms are likely contributing to the anti-tumor effects elicited by the HA PD-1 Ig. First, blockade of the endogenous ligands by HA PD-1 Ig and concomitant elimination of PD-1-mediated inhibitory signals at the effector T cell level may lead to increased T cell proliferation and elevated effector cytokine (e.g., IFN-γ) levels at the tumor site. Second, it has been shown in other models that PD-1 blockade reduces the population of highly immunosuppressive myeloid-derived suppressor cells (MDSC), which inhibit anti-tumor immune responses (John et al., 2013). Third, blockade of PD-1 can decrease immunosuppressive Treg function, as PD-1 signaling promotes Treg development, maintenance and functional response (Francisco et al., 2009).

Intriguingly, administration of HA PD-1 Ig alone did not cause significant difference in tumor growth in this highly invasive, spontaneous metastatic lung tumor model. As indicated tumor immunogenicity is major determinant of responsiveness to immunotherapy. In addition, given the observed variations in the penetrance of autoimmune diseases observed in genetically PD-1 deficient mice, it is likely that host genetic determinants also play an important role. It remains to be determined if monotherapy using HA PD-1 Ig can enhance control of other type of solid tumors, in mice of various genetic backgrounds.

One significant advantage of PD-1 mediated immunotherapy, compared to other checkpoint inhibitors, such as CTLA-4, is the reduced rate of undesirable side effects observed in clinical trials (Hodi et al., 2010, Pardoll, 2012, Robert et al., 2011). This difference is likely due to the primary involvement of PD-1 in the regulation of peripheral effector responses within inflamed tissues and the tumor microenvironment, as opposed to CTLA-4, which primarily regulates the priming phase of the T cell response within the lymph nodes. It is anticipated that HA PD-1 Ig selectively binds to peripheral cells possessing high surface densities of PD-Ligands, such as tumor cells within RT-induced inflammatory environments. This engagement would reduce inhibitory signaling into antigen-specific effector T cells recruited to these sites, and thus enhance the local activation of T cells with specificity for the targeted malignancies.

An important feature of the HA PD-1 Ig is that it is fully human, possessing only a single point mutation, as opposed to humanized mouse monoclonal antibodies that have the tendency to elicit the formation of anti-drug antibodies after repeated treatments, which can decrease clinical efficacy and contribute to treatment failure (Getts et al., 2010). Furthermore, as evidenced by the present studies, the intrinsic cross reactivity of the PD-1 based reagent between species readily supports evaluation in a wide range of preclinical animal models.

In addition to cancer immunotherapy, HA PD-1 Ig may also find utility for the treatment of infectious diseases. Recent studies have shown that enhancement of T cell activation through PD-1 blockade is beneficial for chronic viral infections with HIV, HCV, HBV and HTLV (Kozako et al., 2009, Porichis and Kaufmann, 2012, Watanabe et al., 2010). In these chronic infections, PD-1 is upregulated on virus-specific T cells, which, as a consequence, exhibit an “exhausted phenotype”, and PD-1 blockade leads to re-invigoration of virus-specific effector functions (Barber et al., 2006, Sakthivel et al., 2012). Host responses to other, non-viral pathogens, such as fungi, protozoa, worms and bacteria, have also been shown to be regulated by PD-1 (reviewed in (Sharpe et al., 2007), and therefore could be targeted by manipulating the PD-1 associated pathways. Bacterial (Wu et al., 2010), fungal (Lazar-Molnar et al., 2008a), parasitic (Terrazas et al., 2005), (Smith et al., 2004), and protozoan (Joshi et al., 2009) infections have been shown to induce increased expression of peripheral PD-L1 and PD-L2, which contribute to evasion of the host immune response. Blockade by HA PD-1 Ig would likely reverse the immune suppression induced by these infectious agents, resulting in effector T cells activation and possibly therapeutic benefit.

PD-1 has been an active target for the application of selection strategies to generate engineered reagents with defined biochemical and functional properties. Chang and colleagues exploited mirror-image phage display to generate a protease resistant D-amino acid dodecamer that bound PD-L1 with modest affinity (~ 0.5 μM), and could antagonize the PD-1/PD-L1 interaction *in vitro* and *in vivo* (Chang et al., 2015). Maute and colleagues utilized yeast cell surface display to generate a human PD-1 variant harboring 10 point mutations, which exhibited high affinity for PD-L1 (K_d_ ~ 0.1 nM), but no appreciable interaction with PD-L2 (Maute et al., 2015). This relatively small reagent (molecular weight of ~ 14 kDa reagent) possessed desirable tumor penetration properties, compared to conventional mAbs (molecular weight of ~ 150,000 kDa), and could be used as a PET imaging tracer to distinguish between PD-L1 positive and negative tumors in live mice, and was effective at reducing tumor volume in small and large CT26 syngeneic tumor models in mice in a preclinical murine model of colon cancer. These reports underscore the continued need to develop and evaluate reagents with engineered affinities, selectivities and biophysical properties to treat the broadest range of malignancies.

In summary, using a structure-guided approach, we developed a high-affinity PD-1 Ig fusion protein harboring a single point mutation, which can be used to manipulate the PD-1/PD-Ligand interactions. This bivalent reagent has several features that may contribute to its *in vitro* and *in vivo* immunomodulatory properties and make it an attractive potential therapeutic, including bi-specific binding behavior (i.e., recognition of both PD-L1 and PD-L2) and extensive cross reactivity with the murine PD-Ligands, which greatly facilitates *in vivo* characterization. By demonstrating the synergistic effects of combining HA PD-1 Ig treatment with targeted radiation therapy, our findings provide additional strategies for the therapeutic manipulation of the immunosuppressive tumor microenvironment, increased control of primary and metastatic tumor burden and the establishment of immunological memory against tumor recurrence. These results underscore the potential of rational combination therapies as important future modalities for tumor immunotherapy.

## Conflict of Interest Statement

E. Lazar-Molnar and S.C. Almo have filed a patent on the high-affinity PD-1 Ig. The authors declare no additional competing financial interests.

## Author Contributions

ELM designed and performed the *in vitro* experiments, analyzed the data and interpreted the results. ES contributed to the *in vitro* proliferation studies. LS, TQ and IB performed the *in vivo* tumor experiments and interpreted the results. UAR acquired the crystallography data and provided valuable help with the analysis. EP helped with the protein refolding and purification. SCA, SGN and CG supervised design, data analysis and interpretation. ELM, TQ, LS, CG and SCA wrote the manuscript.

## Figures and Tables

**Table 1:** Human PD-1 mutants and their binding behavior. Structure-based PD-1 mutants were expressed on the surface of HEK293 cells, and binding to PD-L1 and PD-L2 Ig fusion proteins was evaluated by flow cytometry.

Binding behavior	Human PD-1 mutants
Reduced or no binding to both ligands	K78A, I126A^a^
Reduced binding to PD-L1 only	I134A, L128A, L128R, E136A
Increased binding to both PD-L1 and PD-L2	A132L, A132I, A132F, A132T, A132V

a 50% reduced binding to PD-L2.

**Table 2:** Equilibrium dissociation constants (K_d_s) for the interactions between PD-Ligands, and human and mouse PD-1 and their high-affinity A132L mutants. K_d_s were determined by non-linear fitting of SPR data, using immobilized PD-Ligands and soluble monomeric wild type or mutant PD-1. K_d_ values are in μM concentration, followed by the standard errors of the fittings.

	huPD-1	huA132L	mPD-1	mA132L
huPD-L1	6.36 ± 0.57	0.14 ± 0.01	4.02 ± 0.06	0.48 ± 0.02
huPD-L2	0.19 ± 0.019	0.0065 ± 0.0014	10.69 ± 0.83	1.18 ± 0.08
mPD-L1	3.81 ± 0.08	0.23 ± 0.01	5.43 ± 0.08	2.78 ± 0.03
mPD-L2	0.27 ± 0.01	0.029 ± 0.004	2.80 ± 0.15	0.47 ± 0.02

## Appendix A Supplementary data

Supplementary material Image 1
