# Supplementary material for: Structure-guided development of a high-affinity human Programmed Cell Death-1: Implications for tumor immunotherapy
Source: eBioMedicine. 2017 Feb 6;17:30–44. doi: 10.1016/j.ebiom.2017.02.004 (PMC5360572; doi:10.1016/j.ebiom.2017.02.004)
Supplement: Supplementary file 1 — Supplementary material [file mmc1.pdf]

Supplementary Table 1. Crystallographic data collection, phasing and refinement statistics.

Data sets	huPD-1 (A132L)
**PDB ID**	**3RRQ**
**Source**	NSLS X29A
**Data collection**	
Space Group	P 65 2 2
Cell dimension	
a, b, c (Å)	46.02, 46.02, 187.39
α, β, γ (°)	90.0, 90.0, 120.0
Wavelength (Å)	0.979
Resolution (Å)	36.68-2.1
Observed reflections	132630
Unique reflections	7612
Completeness, %	99.9 (100) [1]
R_{merge}	0.066 (0.572) [1]
Average $I/\sigma I$	45.3 (5.8) [1]
**Search probes for molecular replacement**	Mouse PD-1 (1NPU)
**Refinement statistics**	
Resolution (Å)	2.1
No. reflections (work/test)	7526/350
R_{work}/R_{free} (5% of data)	0.214/0.249
**Number of atoms**	
Protein	868
Solvent (H_2O)	35
**RMS deviations**	
Bond length, Å	0.013
Bond angles, °	1.501
**Mean B values, (Å^2)**	48.96
Main Chain	47.96
Side Chain/Water	49.90
**Ramachandran plot (MolProbity)**	
Favored, %	95.3
Allowed, %	97.2
Outliers, %	2.8

[1] Values in parentheses correspond to highest resolution shell.

Supplementary Figure Legends

Supplementary Figure 1. Substitution of A132 to hydrophobic residues in human PD-1 increases binding to both PD-L1 and PD-L2. Wild-type and mutant PD-1 were expressed in HEK293 cells and binding to PD-L1 and PD-L2 Ig was determined by flow cytometry. Data are normalized to wild-type PD-1 binding. Data are representative of three independent experiments.

Supplementary Figure 2. Homology of PD-L1 and PD-L2 sequences from various species. Structural annotations are based on the human PD-L1 (PDB Code) and mouse PD-L2 (PDB code) structures. Asterisks are used to indicate residues in the binding interface, triangles indicated potential N-glycosylation sites.

Supplementary Figure 3. Binding affinities of wild type and A132L mutant human PD-1 to PD-L1 and PD-L2. SPR data and equilibrium dissociation constants between immobilized human PD-L1 and **(A)** soluble wild type, or **(B)** soluble A132L mutant PD-1, as well as immobilized human PD-L2 and **(C)** wild-type or **(D)** A132L mutant PD-1. K_ds are shown in μM, standard errors from data fitting are shown. Data are representative of two independent experiments.

Supplementary Figure 4. Cross-reactivity between mouse and human PD-1 in binding to PD-Ligands. Equilibrium binding dissociation constants were determined by SPR using immobilized mouse PD-L1 and (A) mouse PD-1, or (B) human PD-1; immobilized human PD-L1 and (C) human PD-1 or (D) mouse PD-1; immobilized mouse PD-L2 and (E) mouse PD-1,

or (F) human PD-1, as well as immobilized human PD-L2 and (G) human PD-1, or (H) mouse PD-1. Kd-s are shown in μM, standard errors from Langmuir data fitting are shown.

Supplementary Figure 5. Design of a soluble chimeric high-affinity (HA) PD-1 Ig fusion protein. (A) Overall architecture of the Ig fusion constructs: wild-type or mutant PD-1 ectodomains were fused to the hexa-His-tagged Fc segment of human IgG1. **(B-C)** Binding of wild-type and mutant PD-1 Ig to human PD-L1 **(B)** and PD-L2 **(C)**, and mouse PD-L1 **(D)** and PD-L2 **(E).** Full-length PD-Ligands were expressed in HEK293 cells, binding was detected by flow cytometry. K78A (NB) PD-1 Ig was used as negative control. Data are representative of 3 independent experiments.

Supplementary Figure 6. Phenotypic characteristics of mature human dendritic cells differentiated from monocytes. (A) Increased expression of MHC class II, CD11c, PD-L1, PD-L2, CD83 and B7-1 and lack of expression of CD14 indicates formation of mature dendritic cells from monocytes, upon culture in the presence of GM-CSF and IL-4, and activation with TNF-α. (B) Monocytes are CD11c and CD14 positive, MHC class II, B7-1 and PD-L1 low, PD-L2 and CD83 negative. Representative FSC-SSC plots and gating strategy are shown for each cell type.

Supplementary Figure 7. HA PD-1 Ig dose-dependently increases T cell cytokine secretion after allogeneic activation. Representative data for interferon-γ (A), TNF-α (B), IL-5 (C) and IL-13 (D) are shown. HA PD-1 Ig stimulates cytokine production starting at 0.5 μg/ml, no significant effect was observed for wild-type PD-1 at either 0.5 or 5 μg/ml. Data

from one representative experiment are shown as replicates of four, error bars represent standard errors of mean.

Supplementary Figure 8. 3LL cells express PD-L1, but not PD-L2. 3LL cells were stained with monoclonal antibody to PD-L1 (A) and PD-L2 (B) and analyzed by flow cytometry. Antibody staining is shown in bold histogram, isotype control as regular histogram. Numbers indicate geometric mean fluorescence intensities of specific antibody staining versus isotype control.

Supplementary Figure 9. Flow cytometry analysis of the tumor microenvironment. (**A)** Surface markers on tumor DC detected by flow cytometry, reported as mean fluorescence intensity (MFI). (**B).** Percentage of PD-L1 expression on tumor cells. (**C)** Percentage of tumor infiltrating $CD8^+$ lymphocytes (TIL) analyzed by flow cytometry. Single dots indicate individual mice (n=3-4/group). Data are represented as mean, SD; * $p<0.05$, ** $p<0.01$, *** $p<0.001$ (Student's *t* test).

Suppl Fig 1

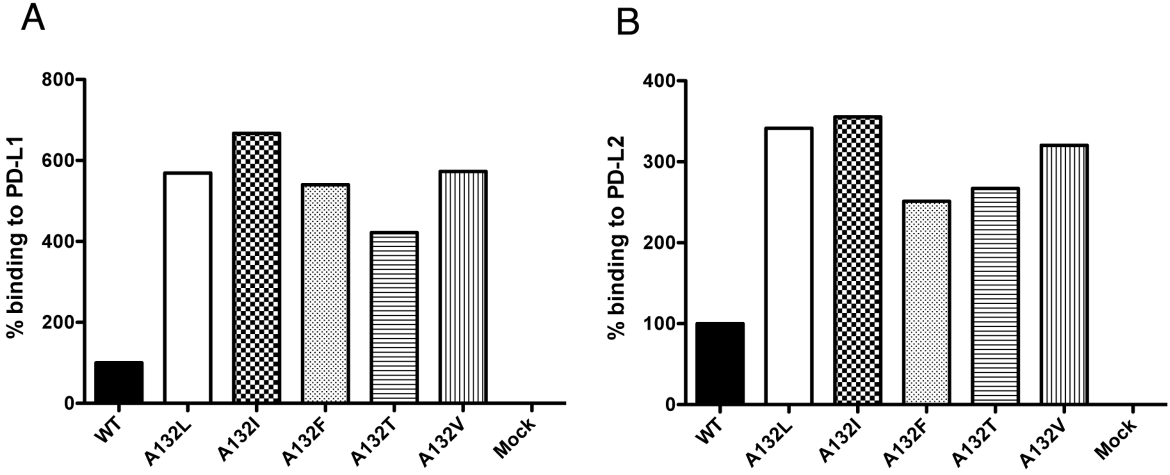
A
B
% binding to PD-L1
% binding to PD-L2
800
600
400
200
0
300
100
WT
A132L
A132I
A132F
A132T
A132V
Mock

Suppl Fig 2

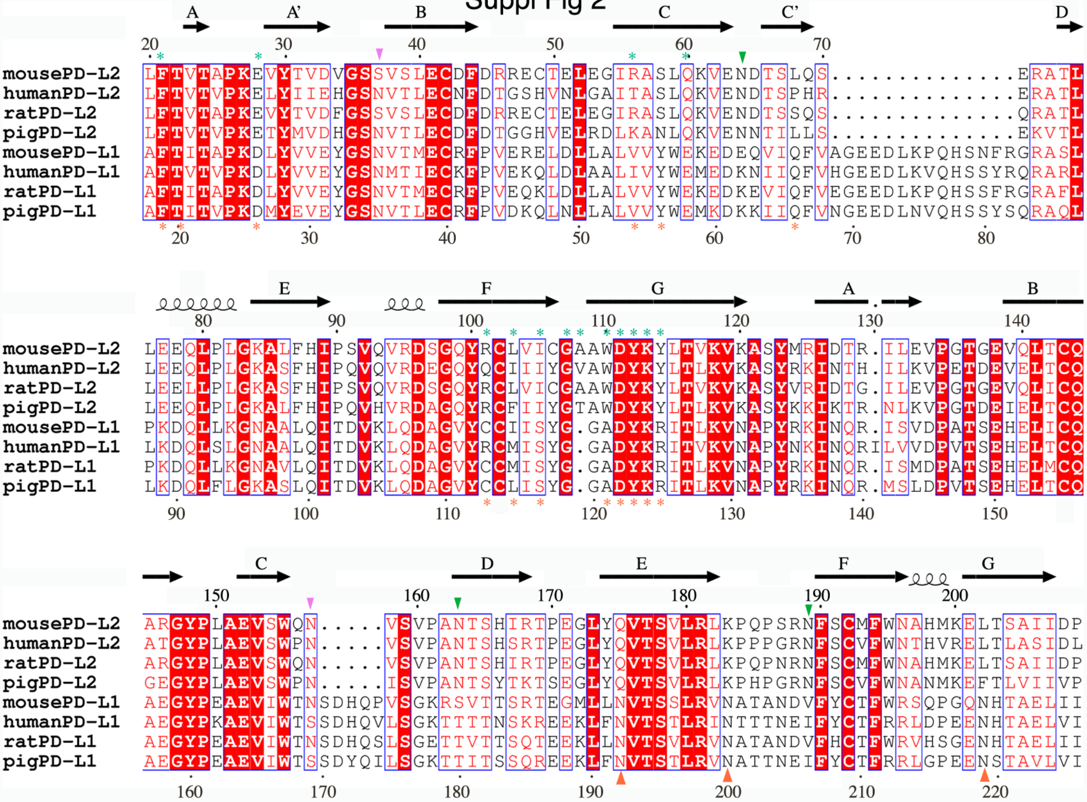
A A' B C C' D
20 30 40 50 60 70
mousePD-L2 LFTVTAPKEVYTVDVGSSVSLECDFDRRECTELEGIRASLQKVENDTSLQS...............ERATL
humanPD-L2 LFTVTVPKELYIIEHGSNVTLECNFDTGSHVNLGAITASLQKVENDTSPHR...............ERATL
ratPD-L2 LFTVTAPKEVYTVDFGSSVSLECDFDRRECTELEGIRASLQKVENDTSSQS...............QRATL
pigPD-L2 LFTVTVPKETYMVDHGSNVTLECDFDTGGHVELRDLKANLQKVENNTILLS...............EKVTL
mousePD-L1 AFTITAPKDLYVVEYGSNVTMECRFPVERELDLLALVVYWEKEDEQVIQFVAGEEDLKPQHSNFRGRASL
humanPD-L1 AFTVTVPKDLYVVEYGSNMTIECKFPVEKQLDLAALIVYWEMEDKNIIQFVHGEEDLKVQHSSYRQRARL
ratPD-L1 AFTITAPKDLYVVEYGSNVTMECRFPVEQKLDLLALVVYWEKEDKEVIQFVEGEEDLKPQHSSFRGRAFL
pigPD-L1 AFTITVPKDMYEVEYGSNVTLECRFPVDKQLNLLALVVYWEMKDKKIIQFVNGEEDLNVQHSSYSQRAQL
20 30 40 50 60 70 80
E F G A B
80 90 100 110 120 130 140
mousePD-L2 LEEQLPLGKALFHIPSVQVRDSGQYRCLVICGAAWDYKYLTVKVKASYMRIDTR.ILEVPGTGEVQLTCQ
humanPD-L2 LEEQLPLGKASFHIPQVQVRDEGQYQCIIIYGVAWDYKYLTLKVKASYRKINTH.ILKVPETDEVELTCQ
ratPD-L2 LEELLPLGKASFHIPSVQVRDSGQYRCLVICGAAWDYKYLTVKVKASYVRIDTG.ILEVPGTGEVQLICQ
pigPD-L2 LEEQLPLGKALFHIPQVHVRDAGQYRCFIIYGTAWDYKYLTLKVKASYKKIKTR.NLKVPGTDEIELTCQ
mousePD-L1 PKDQLLKGNAALQITDVKLQDAGVYCCIISYG.GADYKRITLKVNAPYRKINQR.ISVDPATSEHELICQ
humanPD-L1 LKDQLSLGNAALQITDVKLQDAGVYRCMISYG.GADYKRITVKVNAPYNKINQRILVVDPVTSEHELTCQ
ratPD-L1 PKDQLLKGNAVLQITDVKLQDAGVYCCMISYG.GADYKRITLKVNAPYRKINQR.ISMDPATSEHELMCQ
pigPD-L1 LKDQLFLGKASLQITDVKLQDAGVYCCLISYG.GADYKRITLKVNAPYRKINQR.MSLDPVTSEHELTCQ
90 100 110 120 130 140 150
C D E F G
150 160 170 180 190 200
mousePD-L2 ARGYPLAEVSWQN.....VSVPANTSHIRTPEGLYQVTSVLRLKPQPSRNFSCMFWNAHMKELTSAIIDP
humanPD-L2 ATGYPLAEVSWPN.....VSVPANTSHSRTPEGLYQVTSVLRLKPPPGRNFSCVFWNTHVRELTLASIDL
ratPD-L2 ARGYPLAEVSWQN.....VSVPANTSHIRTPEGLYQVTSVLRLKPQPNRNFSCMFWNAHMKELTSAIIDP
pigPD-L2 GEGYPLAEVSWPN.....ISVPANTSYTKTSEGLYQVTSVLRLKPHPGRNFSCVFWNANMKEFTLVIIVP
mousePD-L1 AEGYPEAEVIWTNSDHQPVSGKRSVTTSRTEGMLLNVTSSLRVNATANDVFYCTFWRSQPGQNHTAELII
humanPD-L1 AEGYPKAEVIWTSSDHQVLSGKTTTTNSKREEKLFNVTSTLRINTTTNEIFYCTFRRLDPEENHTAELVI
ratPD-L1 AEGYPEAEVIWTNSDHQSLSGETTVTTSQTEEKLLNVTSVLRVNATANDVFHCTFWRVHSGENHTAELII
pigPD-L1 AEGYPEAEVIWTSSDYQILSGKTTITSSQREEKLFNVTSTLRVNATTNEIFYCTFRRLGPEENSTAVLVI
160 170 180 190 200 210 220

Suppl Fig 3

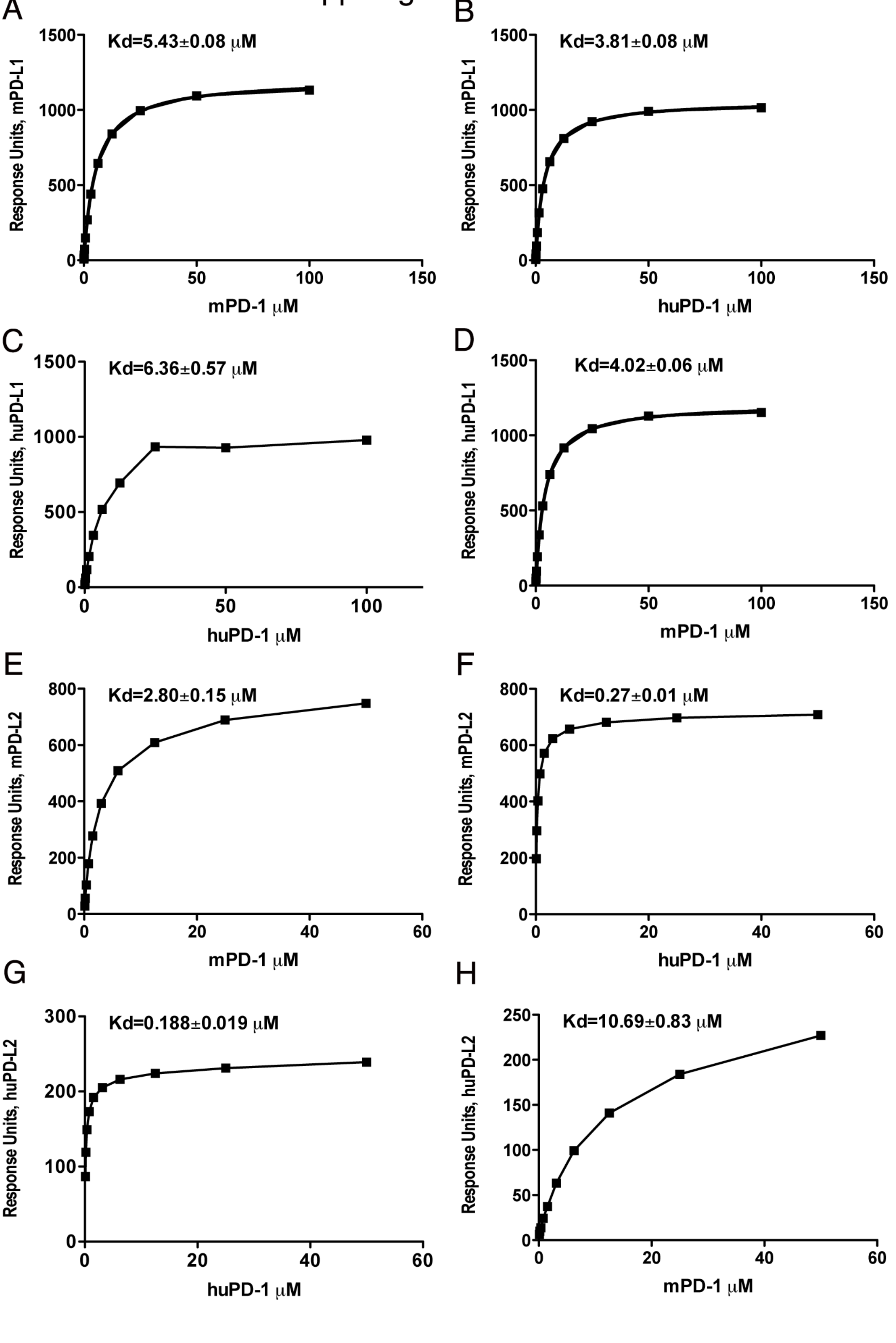
A
Kd=5.43±0.08 μM
Response Units, mPD-L1
mPD-1 μM
B
Kd=3.81±0.08 μM
Response Units, mPD-L1
huPD-1 μM
C
Kd=6.36±0.57 μM
Response Units, huPD-L1
huPD-1 μM
D
Kd=4.02±0.06 μM
Response Units, huPD-L1
mPD-1 μM
E
Kd=2.80±0.15 μM
Response Units, mPD-L2
mPD-1 μM
F
Kd=0.27±0.01 μM
Response Units, mPD-L2
huPD-1 μM
G
Kd=0.188±0.019 μM
Response Units, huPD-L2
huPD-1 μM
H
Kd=10.69±0.83 μM
Response Units, huPD-L2
mPD-1 μM

Suppl Fig 4

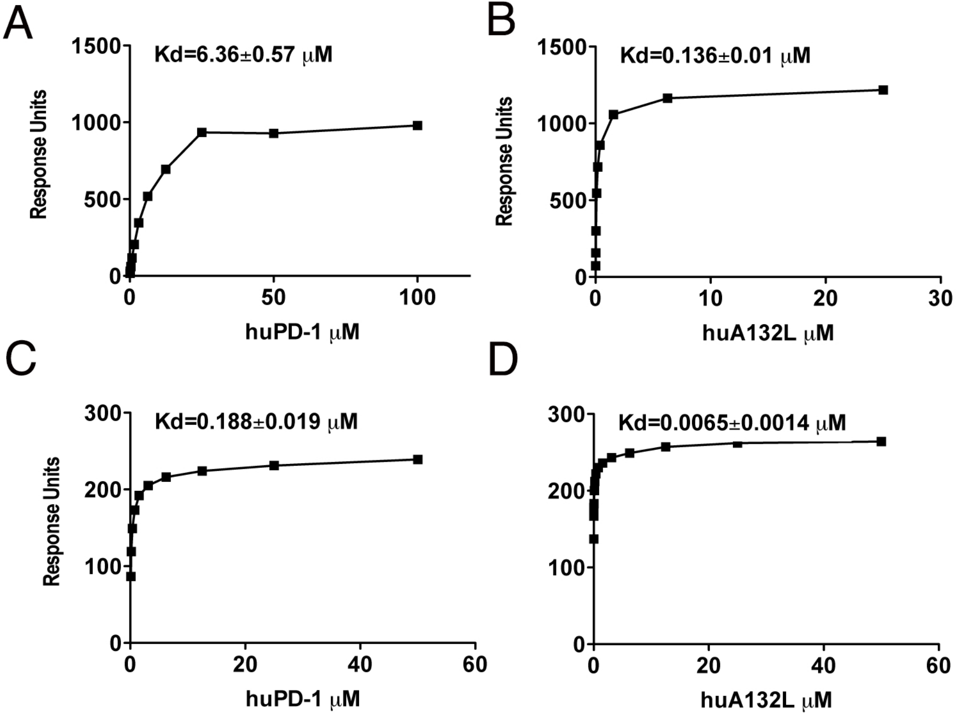
A
Kd=6.36±0.57 μM
Response Units
1500
1000
500
0
0
50
100
huPD-1 μM
B
Kd=0.136±0.01 μM
Response Units
1500
1000
500
0
0
10
20
30
huA132L μM
C
Kd=0.188±0.019 μM
Response Units
300
200
100
0
0
20
40
60
huPD-1 μM
D
Kd=0.0065±0.0014 μM
300
200
100
0
0
20
40
60
huA132L μM

Suppl Fig 5

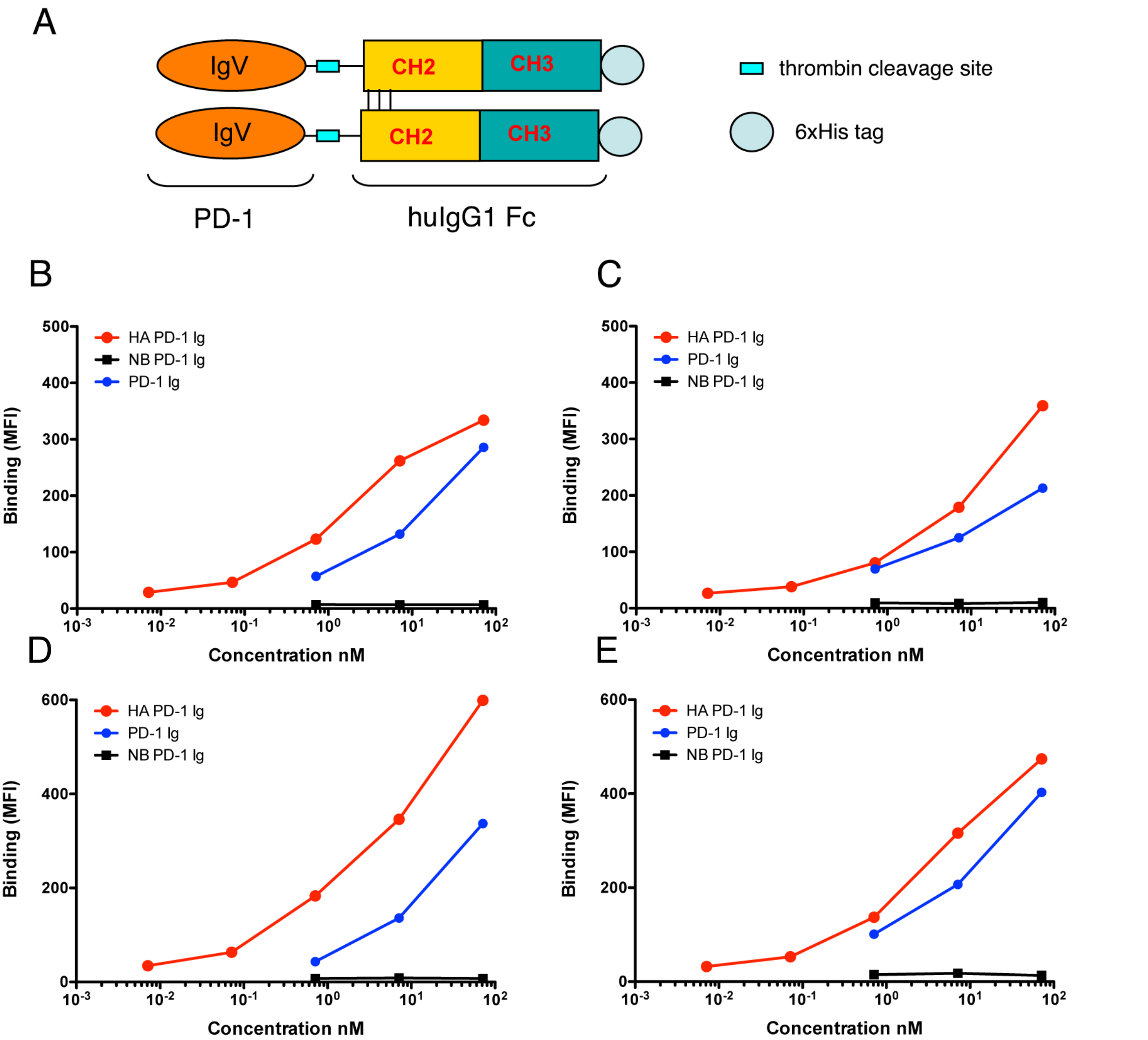
A
IgV
CH2
CH3
IgV
CH2
CH3
PD-1
huIgG1 Fc
thrombin cleavage site
6xHis tag
B
HA PD-1 Ig
NB PD-1 Ig
PD-1 Ig
Binding (MFI)
Concentration nM
C
HA PD-1 Ig
PD-1 Ig
NB PD-1 Ig
Binding (MFI)
Concentration nM
D
HA PD-1 Ig
PD-1 Ig
NB PD-1 Ig
Binding (MFI)
Concentration nM
E
HA PD-1 Ig
PD-1 Ig
NB PD-1 Ig
Binding (MFI)
Concentration nM

Suppl Fig 6

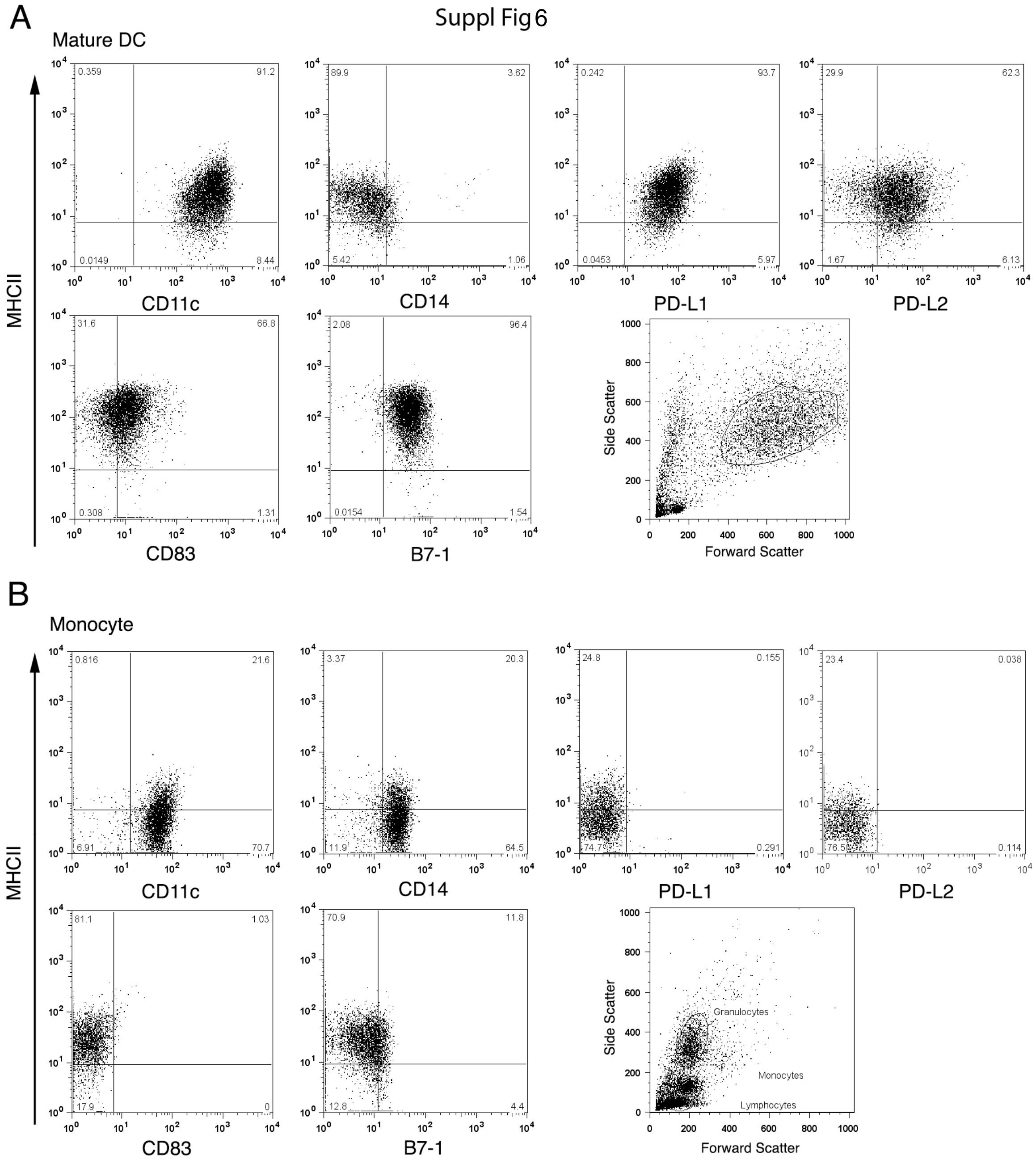
A
Mature DC
MHCII
CD11c
CD14
PD-L1
PD-L2
CD83
B7-1
Side Scatter
Forward Scatter
B
Monocyte
MHCII
CD11c
CD14
PD-L1
PD-L2
CD83
B7-1
Side Scatter
Forward Scatter
Granulocytes
Monocytes
Lymphocytes

Suppl Fig 7

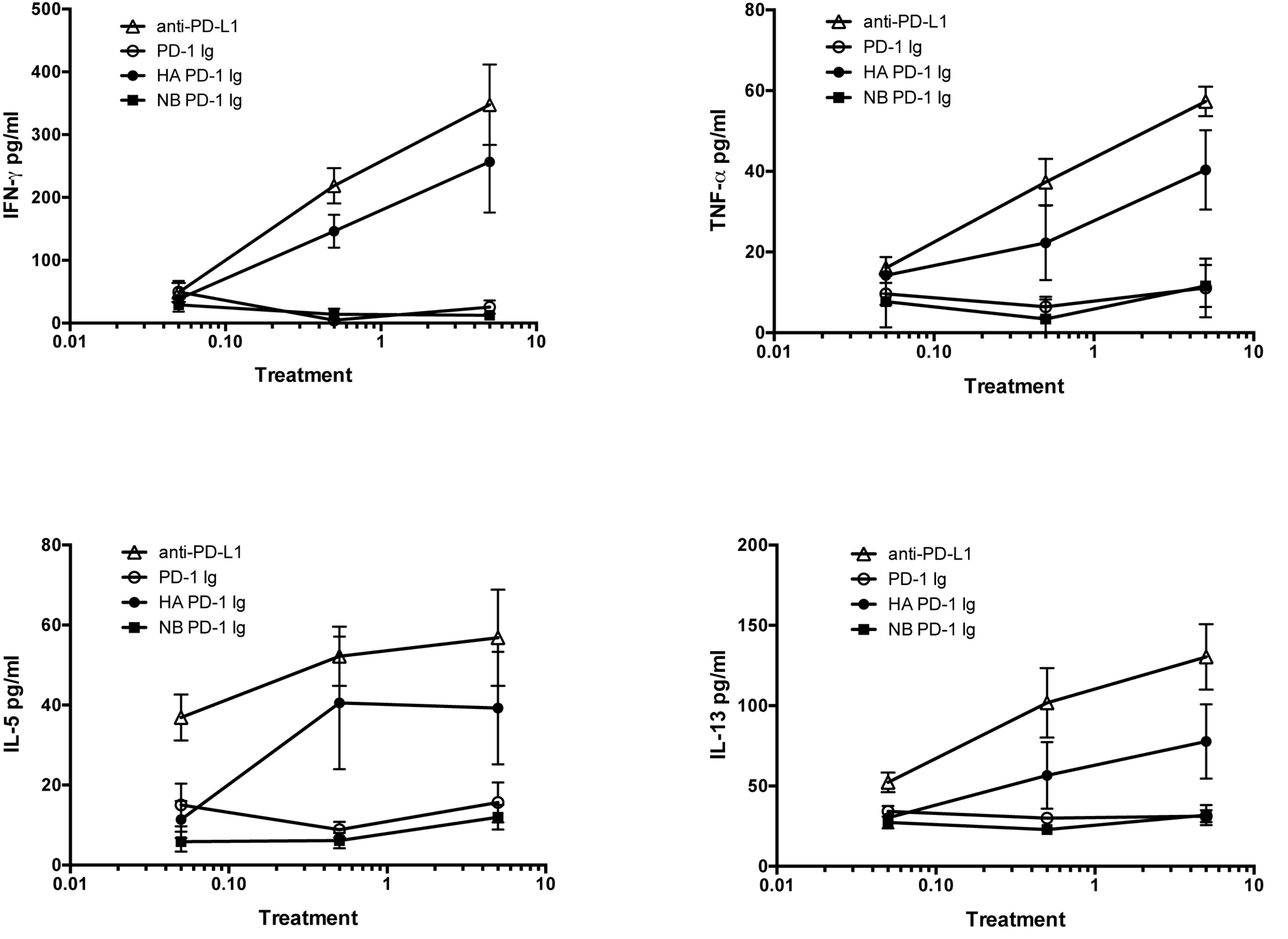
anti-PD-L1
PD-1 Ig
HA PD-1 Ig
NB PD-1 Ig
IFN-γ pg/ml
TNF-α pg/ml
IL-5 pg/ml
IL-13 pg/ml
Treatment
0.01
0.10
1
10

Suppl Fig 8

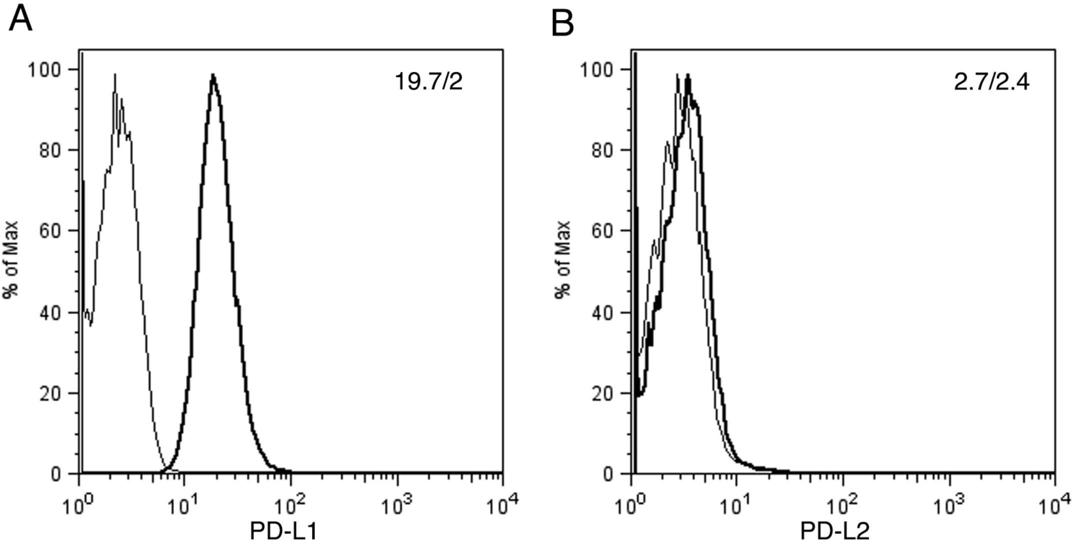
A
B
100
80
60
40
20
0
% of Max
19.7/2
2.7/2.4
10^0
10^1
10^2
10^3
10^4
PD-L1
PD-L2

Suppl Fig 9

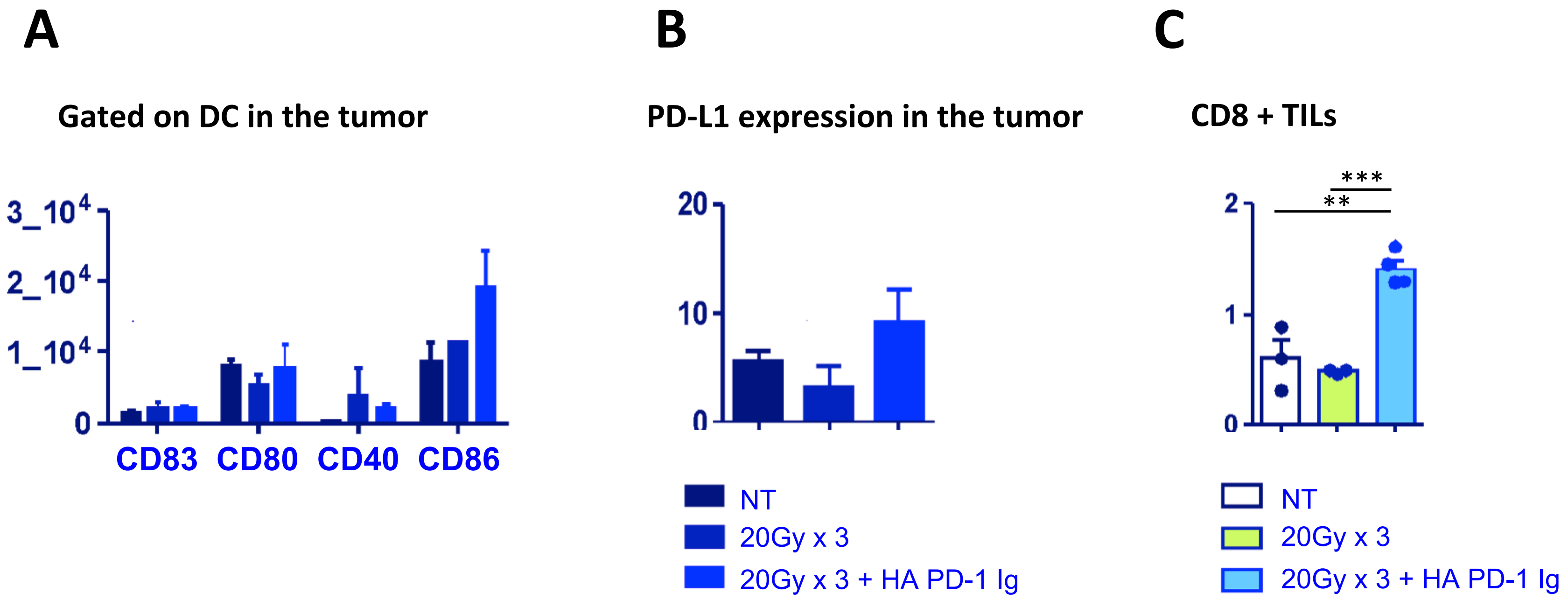
A
Gated on DC in the tumor
3_10⁴
2_10⁴
1_10⁴
0
CD83
CD80
CD40
CD86
B
PD-L1 expression in the tumor
20
10
0
NT
20Gy x 3
20Gy x 3 + HA PD-1 Ig
C
CD8 + TILs
***
**
2
1
0
NT
20Gy x 3
20Gy x 3 + HA PD-1 Ig
